# Cell‐Like Synthetic Supramolecular Soft Materials Realized in Multicomponent, Non‐/Out‐of‐Equilibrium Dynamic Systems

**DOI:** 10.1002/advs.202306830

**Published:** 2023-11-28

**Authors:** Ryou Kubota, Itaru Hamachi

**Affiliations:** ^1^ Department of Synthetic Chemistry and Biological Chemistry Graduate School of Engineering Kyoto University Katsura Nishikyo‐ku Kyoto 615‐8510 Japan; ^2^ JST‐ERATO Hamachi Innovative Molecular Technology for Neuroscience Kyoto University Nishikyo‐ku Katsura 615‐8530 Japan

**Keywords:** coacervate, hydrogel, multicomponent, nonequilibrium, spatiotemporal patterning, supramolecular chemistry, systems chemistry

## Abstract

Living cells are complex, nonequilibrium supramolecular systems capable of independently and/or cooperatively integrating multiple bio‐supramolecules to execute intricate physiological functions that cannot be accomplished by individual biomolecules. These biological design strategies offer valuable insights for the development of synthetic supramolecular systems with spatially controlled hierarchical structures, which, importantly, exhibit cell‐like responses and functions. The next grand challenge in supramolecular chemistry is to control the organization of multiple types of supramolecules in a single system, thus integrating the functions of these supramolecules in an orthogonal and/or cooperative manner. In this perspective, the recent progress in constructing multicomponent supramolecular soft materials through the hybridization of supramolecules, such as self‐assembled nanofibers/gels and coacervates, with other functional molecules, including polymer gels and enzymes is highlighted. Moreover, results show that these materials exhibit bioinspired responses to stimuli, such as bidirectional rheological responses of supramolecular double‐network hydrogels, temporal stimulus pattern‐dependent responses of synthetic coacervates, and 3D hydrogel patterning in response to reaction–diffusion processes are presented. Autonomous active soft materials with cell‐like responses and spatially controlled structures hold promise for diverse applications, including soft robotics with directional motion, point‐of‐care disease diagnosis, and tissue regeneration.

## Graphical abstract

The next challenge in supramolecular chemistry is the controlled non‐/out‐of‐equilibrium organization of multiple types of supramolecules within a single system, like living cells. This perspective highlights the achievements in bioinspired stimulus responses and 3D hydrogel patterning by integration of multiple synthetic supramolecules and other functional materials in an orthogonal and/or cooperative manner.

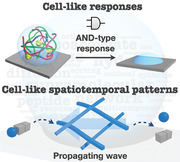

## 1 Introduction

Living cells are multicomponent, nonequilibrium supramolecular systems composed of a myriad of bio‐supramolecules, including proteins, (deoxy)ribonucleic acids (DNA/RNA), lipids, and metabolites.^[^ ^1^ ^]^ Living cells can integrate multiple bio‐supramolecules independently and cooperatively to execute elaborate physiological functions, including metabolism, motility, immune responses, and neural activity, which cannot be achieved by individual biomolecules. For example, the assembly of actin and tubulin monomers gives rise to orthogonal supramolecular fibers, actin filaments, and microtubules, which exhibit distinct roles in live cells.^[^ ^2^, ^3^, ^4^ ^]^ Orthogonal coupling of cytoskeletons with lipid membrane enables cells to rationally execute distinct physiological outcomes, including motility, migration, transportation, and mitosis, without mutual interference. In contrast, crosstalk among individual bio‐supramolecules through covalent or noncovalent interactions contributes to the emergence of sophisticated synergistic responses. In intracellular signal transduction systems, such as the mitogen‐activated kinase cascade, a series of phosphoproteins arranged along a pathway transmit signals in a sequential, domino‐like manner. Kinase cascades effectively amplify signals through reaction and diffusion processes.^[^ ^1^, ^5^ ^]^ Another intriguing example is the autonomous formation of static and dynamic patterns, such as animal body patterns and embryonic development, realized by the interplay among multiple bio‐supramolecules via the reaction–diffusion mechanism.^[^ ^6^, ^7^, ^8^, ^9^, ^10^ ^]^ In bacterial cells, the spatial position of cell division is precisely determined by the oscillation of the Min protein complex between cell poles, which is driven by the assembly and disassembly of the protein complex coupled with its diffusion across the bacterial cells.^[^ ^11^ ^]^ These biological design strategies provide valuable clues for the development of synthetic supramolecular systems with cell‐like responses, functions, and spatially controlled hierarchical structures.

**Figure 1:**
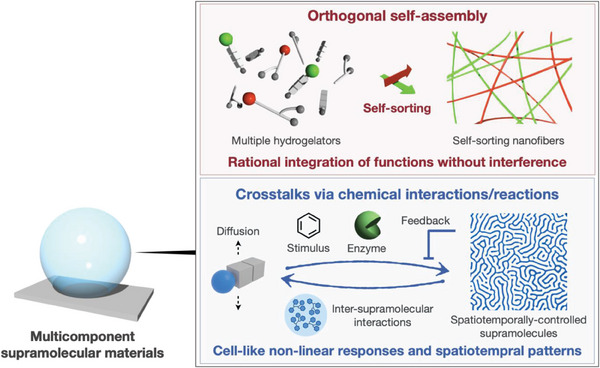
Schematic illustration of the research concept.

Supramolecular chemistry is one of the most active research fields and has advanced considerably by mimicking biological molecules.^[^ ^12^, ^13^, ^14^ ^]^ To date, biological molecules have inspired the construction of numerous types of synthetic supramolecules, including host–guest chemistry‐based artificial enzymes,^[^ ^15^, ^16^, ^17^ ^]^ metal–organic cages and frameworks with enzyme‐mimetic nano‐sized spaces,^[^ ^18^, ^19^, ^20^, ^21^, ^22^ ^]^ vesicles and coacervates for compartmentalization,^[^ ^23^, ^24^, ^25^, ^26^, ^27^, ^28^ ^]^ and nanofibers and gels resembling cytoskeletons and the extracellular matrix.^[^ ^13^, ^29^ ^]^ Although the assembly of individual molecules has been extensively investigated, the interplay and networking among supramolecules remain at the early stage of investigation. The next grand challenge in supramolecular chemistry is thus to control the organization of multiple types of supramolecules in a single system to realize emergent properties and functions in an orthogonal and/or cooperative manner.^[^ ^30^, ^31^, ^32^, ^33^, ^34^, ^35^ ^]^ These approaches would enable the development of next‐generation smart materials that are hierarchically structured with anisotropic arrangements of multiple supramolecules, thus allowing unique nonlinear responses to stimuli and cell‐like signal amplification. To realize this systems chemistry approach, supramolecular chemists now face the challenge of developing methods for constructing multiple types of supramolecules in an orthogonal manner, especially in an aqueous environment; regulating interactions among the orthogonal supramolecules; and integrating chemical and enzymatic reactions to prevent the interference of reactive functional groups. In this perspective, we describe our recent progress in constructing multicomponent supramolecular soft materials through the hybridization of supramolecules, such as self‐assembled nanofibers, gels, and coacervates, with other functional molecules, including polymer gels and enzymes (**Figure** **1**). Moreover, we present results showing that these materials exhibit bioinspired responses to stimuli, such as bidirectional rheological responses of supramolecular double‐network hydrogels, temporal stimulus pattern‐dependent responses of synthetic coacervates, and 3D hydrogel patterning in response to reaction–diffusion processes. Other reviews that also offer insights into constructing supramolecular soft materials are available.^[^ ^36^, ^37^, ^38^, ^39^, ^40^, ^41^, ^42^ ^]^

## 2 General Design of Building Blocks and Self‐Assembly Strategies for Multicomponent and Non‐/Out‐of‐Equilibrium Dynamic Systems

**Figure 2:**
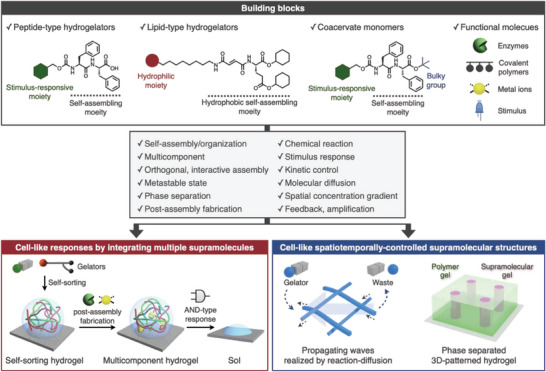
Schematic illustration of cell‐like responses and spatiotemporally‐controlled structures realized through the key concepts of self‐assembly with multiple building blocks.

In this section, we highlight the structural design of building blocks for supramolecular hydrogels and coacervates described in this Perspective (**Figure** **2** top). Stimuli‐responsive peptide‐type hydrogelators of di‐ or tri‐phenylalanine which have been extensively used as a self‐assembling moiety in aqueous solutions^[^ ^43^, ^44^, ^45^, ^46^, ^47^, ^48^ ^]^ are one of the key players. The peptide‐type hydrogelators form into crystalline nanofibers with a *β*‐sheet like structure, mainly driven by *π*–*π* interactions between benzene rings and hydrogen bonds between amide groups. We previously discovered the addition and deletion of the hydrophobic moiety at the *N*‐terminus of these peptides through chemical reactions can induce the sol‐to‐gel and gel‐to‐sol transitions, respectively.^[^ ^33^, ^49^, ^50^ ^]^ Lipid‐type hydrogelators, another key player, are amphiphiles that have a hydrophilic head group (e.g., phosphate and *N*‐acetylgalactosamine, GalNAc) and two cycloalkyl groups as hydrophobic tails.^[^ ^51^, ^52^ ^]^ Due to the too‐short hydrophobic tails, the lipid‐type hydrogelators can't form spherical vesicles, but instead, form 1D nanofibers mainly through hydrophobic interactions between the cycloalkyl chains and hydrogen bonds between fumaramide groups. The interaction modes are remarkably different from those of peptide‐type hydrogelators. Also, we recently developed a new structural motif, tert‐butyl diphenylalaninate (FF‐OtBu) to generate liquid–liquid phase separation (LLPS).^[^ ^53^, ^54^ ^]^ A bulky tBu group newly introduced at the *C*‐terminus of a diphenylalanine peptide may suppress β‐sheet‐based nanofiber formation, and induce LLPS. Unlike the peptide‐type hydrogelators, several types of hydrophilic functional groups are attached at the *N*‐terminus of the FF‐OtBu motif and we have found unique coacervates composed of a short peptide. Furthermore, we integrated other functional molecules, including enzymes (reaction catalysts), synthetic covalent polymers (stiff structure motif), porous materials (molecular recognition), and metal ions (coordination bonding), into supramolecular hydrogels and coacervates, by which sophisticated stimulus‐responsive systems are fabricated.

To realize cell‐like responses, functions, and hierarchical structures using these building blocks, we employed the four self‐assembly strategies (Figure 2 middle, bottom). The first involves the orthogonal self‐assembly of multiple supramolecules through self‐sorting phenomena. We demonstrated that peptide‐ and lipid‐type hydrogelators self‐assemble orthogonally without mixing each other, resulting in the formation of self‐sorting double‐network (SDN) hydrogels. The self‐sorting phenomena allow the rational integration of stimulus responses from these peptide‐ and lipid‐type nanofibers without mutual interference (Section 3.1). Four control factors were identified to date for self‐sorting: i) distinct chemical structures and driving forces of self‐assembly, ii) the same charge (in our case, anionic peptide‐ and lipid‐type hydrogelators were used), iii) the moderate hydrophobicity of the peptide‐type hydrogelators, iv) distinct formation kinetics (lipid‐type hydrogelators self‐assemble first). The second strategy relies on post‐assembly fabrication (PAF) to extend the range of stimulus responses in SDN hydrogels by the addition of other functional molecules (Section 3.2). To retain the self‐sorting network, the functional molecules are implemented after the construction of SDN hydrogels. The PAF strategy is also applicable to in situ degradation and formation of peptide‐type nanofibers in the presence of lipid‐type nanofibers, which enables control of the self‐sorting network (Section 4.2). The third strategy is based on a reaction–diffusion system, which is a widely‐used method to spontaneously generate spatiotemporal patterns. In this Perspective, we describe the emergence of propagating waves of supramolecular nanofibers, whose generation and degradation are controlled under concentration gradients of distinct chemical stimuli (Section 4.1). The interplay between radical polymerization and its inhibition reaction results in temporal stimulus pattern‐dependent responses in synthetic coacervates (Section 3.4). The last employs microscopic/macroscopic phase separation, which is a potent approach for the development of hierarchically structured soft materials. In particular, phase separation enables the construction of 3D patterned hierarchical hydrogel materials through the combination of top‐down fabrication methods (Section 4.4).

## 3 Emergence of Unique Cell‐Like Responses by Incorporating Multiple Supramolecules

This section describes synthetic systems exhibiting unique responses to stimuli, which are obtained by integrating multiple supramolecules and/or other functional molecules in an orthogonal and/or cooperative manner. As an early example, van Esch and coworkers demonstrated the orthogonal self‐assembly of surfactants (micelles and vesicles) and low‐molecular weight (LMW) hydrogelators composed of di‐ or tetraethylene glycol‐modified 1,3,5‐cyclohexyltricarboxamide, which was confirmed with differential scanning calorimetry and cryo‐transmission electron microscopy measurements.^[^ ^55^, ^56^, ^57^ ^]^ Subsequently, they developed a platform by incorporating thermosensitive liposomes encapsulating enzymes to release the small organic molecule covalently attached to the enzyme‐responsive hydrogelator of this orthogonal self‐assembly system.^[^ ^58^ ^]^ Controlled heating of this three‐component system liberates the enzyme into the gel matrix, and subsequent hydrolysis of the hydrogelator releases the small molecule with a release rate controlled by the heating time. We constructed a hydrogel system capable of fluorescence sensing of biologically relevant polyanions and polycations by integrating porous inorganic materials (montmorillonite and mesoporous silica particles (MCM41), respectively) with lipid‐type supramolecular fibers, which allows visualization of analyte‐induced exchange and location shift of fluorescent dyes in this multicomponent hydrogel system.^[^ ^59^, ^60^ ^]^ These pioneering examples indicate that integrating multiple supramolecules can be applied to diagnostic disease‐sensing devices and controlled drug‐release systems.

### 3.1 Bidirectional Rheological Responses of Self‐Sorting Supramolecular Nanofibers

**Figure 3:**
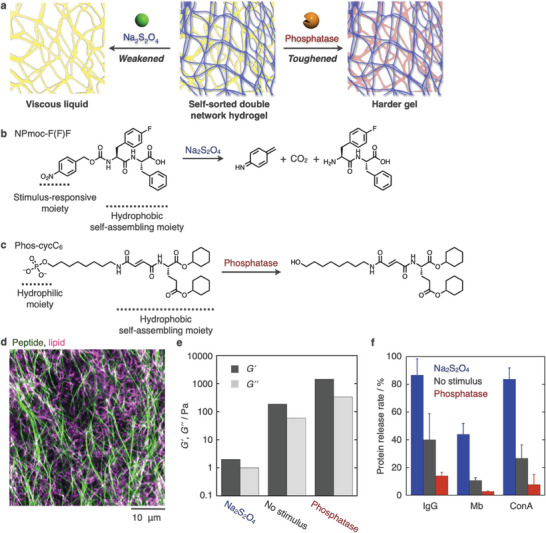
a) Schematic illustration of bidirectional rheological responses of a self‐sorting double network (SDN) hydrogel. b, c) Chemical structures and stimulus responses of (b) a peptide‐type gelator, NPmoc‐F(F)F, and (c) a lipid‐type hydrogelator, Phos‐cycC_6_. d) Confocal image of the self‐sorting double network hydrogel. Green: NPmoc‐F(F)F nanofibers, magenta: Phos‐cycC_6_ nanofibers. e) Rheological analysis of the SDN hydrogels (middle) before and after addition of (left) Na_2_S_2_O_4_ and (right) alkaline phosphatase. f) Release rate of embedded proteins from the SDN hydrogels (black) before and after the addition of (blue) Na_2_S_2_O_4_ and (red) alkaline phosphatase. Mb: myoglobin, ConA: concanavalin A.

Cytoskeletons, such as actin filaments and microtubules, exhibit self‐sorting, which can be applied as a promising approach to rationally integrate supramolecules into a single synthetic system.^[^ ^51^, ^61^, ^62^, ^63^, ^64^, ^65^, ^66^, ^67^, ^68^, ^69^, ^70^, ^71^, ^72^, ^73^, ^74^, ^75^, ^76^, ^77^, ^78^, ^79^, ^80^, ^81^, ^82^, ^83^, ^84^ ^]^ To demonstrate this approach, we constructed SDN hydrogels with bidirectionally tunable rheological properties (**Figure** **3**a).^[^ ^77^ ^]^ Previously, we found that peptide‐ and lipid‐type hydrogelators are promising pairs to achieve self‐sorting in an aqueous environment because of their distinct chemical structures and molecular interactions.^[^ ^73^, ^75^ ^]^ To prepare SDN hydrogels, NPmoc‐F(F)F and Phos‐cycC_6_ were employed as peptide‐ and lipid‐type hydrogelators, respectively (Figure 3b,c). NPmoc‐F(F)F nanofibers decompose in the presence of a reductant (sodium dithionite) through nitrobenzyl group reduction followed by 1,6‐elimination to produce a more hydrophilic F(F)F dipeptide (Figure 3b). In contrast, Phos‐cycC_6_ converts into a more hydrophobic HO‐cycC_6_ in response to the alkaline phosphatase (AP) enzyme to become a stiffer hydrogel (Figure 3c). Finally, these two nanofibers integrate through self‐sorting (Figure 3d). Rheological analysis showed that the resultant self‐sorting hydrogel exhibits bidirectional responses. Specifically, the storage modulus decreased from 187 to 2 Pa after treatment with the reductant but increased to 1450 Pa after the addition of AP (Figure 3e). High‐performance liquid chromatography (HPLC) analysis confirmed that the reductant and AP selectively react with the corresponding hydrogelators. In addition, we succeeded in controlling the release rate of proteins embedded in the SDN hydrogels (Figure 3f). For example, the IgG antibody was slowly released from nontreated self‐sorting hydrogels with a release rate of 40 ± 12% over 10 h, which was bidirectionally changed to 87 ± 19% and 14 ± 2% after treatment with the reductant and AP, respectively. It is clear that self‐sorting can be applied to the design of smart soft materials that are responsive to multiple stimuli for controlled drug release and cell culturing.

### 3.2 Extension of Stimulus Responses of Self‐Sorting Supramolecular Hydrogels by Post‐Assembly Fabrication

**Figure 4:**
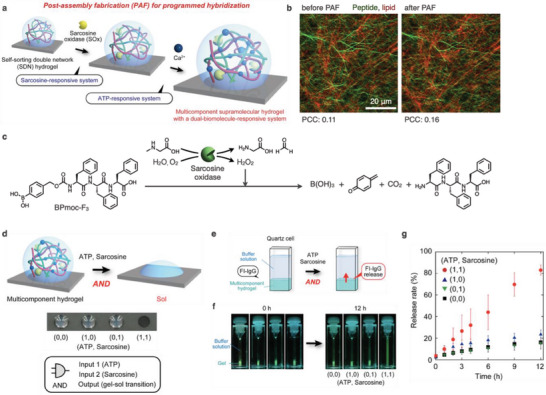
a) Schematic illustration of post‐assembly fabrication (PAF) of an SDN hydrogel. b) Confocal images of the SDN hydrogels of BPmoc‐F_3_ and Phos‐cycC_6_ (left) before and (right) after PAF with sarcosine oxidase and Ca^2+^ ions. PCC: Pearson's correlaion coefficient. c) Stimulus response of BPmoc‐F_3_ through enzymatic generation of H_2_O_2_. d) AND‐type gel‐to‐sol response of the multicomponent SDN hydrogel by addition of sarcosine and ATP. e) Schematic illustration of controlled release of embedded fluorescein‐modified IgG (FL‐IgG) through the AND‐type gel‐to‐sol response. f) Photographs of the multicomponent SDN hydrogels containing FL‐IgG before and after the addition of sarcosine and ATP. g) Release profiles of FL‐IgG from the multicomponent SDN hydrogels.

Although constructing SDN hydrogels by self‐sorting is a powerful approach, it requires delicately balancing the noncovalent interactions of hydrogelators. Therefore, there is currently a limited variety of SDN hydrogels. To extend SDN hydrogels, we proposed a PAF strategy, in which functional molecules and/or enzymes are added to the constructed SDN hydrogel (**Figure** **4**a).^[^ ^78^ ^]^ Sarcosine oxidase (SOx) enzyme and Ca^2+^ ions were selected for the PAF of the BPmoc‐F_3_/Phos‐cycC_6_ SDN hydrogel. SOx generates H_2_O_2_, glycine, and formaldehyde from sarcosine, and generated H_2_O_2_ decomposes BPmoc‐F_3_ nanofibers (Figure 4c). In contrast, Ca^2+^ ions interact with the phosphate groups on the surface of Phos‐cycC_6_ nanofibers to induce a sol‐to‐gel transition through multivalent coordination bonding (Phos‐cycC_6_ forms nanofibers in the solution state). Ca^2+^‐bound Phos‐cycC_6_ nanofibers respond to ATP because Ca^2+^ ions have a stronger affinity for the triphosphate group of ATP than Phos‐cycC_6_, leading to a gel‐to‐sol transition. Overall, the multicomponent SDN hydrogel prepared with PAF using SOx and Ca^2+^ ions exhibits an AND‐type gel‐to‐sol response by the action of biologically relevant molecules, sarcosine and ATP. Confocal imaging^[^ ^85^, ^86^ ^]^ showed that the self‐sorting network is retained even after the PAF process (Figure 4b). The resultant multicomponent SDN hydrogel undergoes a macroscopic gel‐to‐sol transition in response to both sarcosine and ATP, while the hydrogel maintains its gel state following the action of either sarcosine or ATP (Figure 4d). The multicomponent SDN hydrogel is a suitable AND‐type active matrix for the controlled release of fluorescently modified IgG antibodies (Figure 4e–g). Notably, the one‐step mixing protocol, in which Ca^2+^ ions are mixed with a mixture of two hydrogelators before SDN formation, produces a white suspension instead of the SDN hydrogel, indicating that PAF is an essential step in the construction of multicomponent SDN hydrogels with extended stimulus responses.

### 3.3 Nonenzymatic Protein‐Dependent Responses of Multicomponent Supramolecular–Polymer Composite Hydrogels

**Figure 5:**
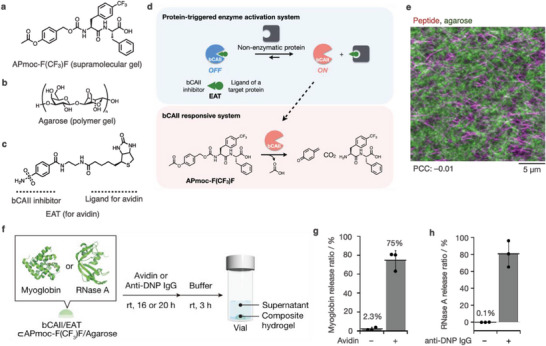
a–c) Chemical structures of (a) an enzyme‐responsive peptide‐type hydrogelator, APmoc‐F(CF_3_)F, (b) agarose, and (c) an EAT molecule for avidin. d) Mechanism of enzyme‐mediated response toward nonenzymatic proteins. e) Confocal microscopic image of a composite hydrogel of APmoc‐F(CF_3_)F and agarose. Magenta: APmoc‐F(CF_3_)F nanofibers, green: agarose networks. f) Experimental protocol for controlled release of proteins embedded in the composite hydrogel containing the EAT system, in response to nonenzymatic proteins. g, h) Release rate of (g) myoglobin and (h) RNase A in response to (g) avidin and (h) anti‐DNP IgG.

In addition to enzymes, nonenzymatic proteins, such as antibodies, often serve as biomarkers for several diseases. However, supramolecular systems that are responsive to nonenzymatic proteins are challenging to develop,^[^ ^87^, ^88^, ^89^, ^90^ ^]^ whereas a variety of supramolecules responsive to enzymes and their produced reactive species have been extensively developed thus far.^[^ ^33^, ^39^, ^40^, ^91^, ^92^, ^93^, ^94^ ^]^ We successfully prepared a supramolecular–polymer composite hydrogel that can respond to nonenzymatic proteins by integrating an enzyme‐responsive supramolecular hydrogelator with an enzyme‐activation system.^[^ ^95^ ^]^ In particular, we designed an enzyme‐responsive hydrogelator, APmoc‐F(CF_3_)F, with an acetoxybenzyl group at the *N*‐terminus (**Figure** **5**a). APmoc‐F(CF_3_)F undergoes a gel‐to‐sol transition in response to bovine carbonic anhydrase II (bCAII). To elicit a response to nonenzymatic proteins, we designed an enzyme‐activation trigger (EAT) molecule consisting of a bCAII inhibitor (benzenesulfonamide) and a ligand of the target nonenzymatic protein connected by a short linker (Figure 5c).^[^ ^96^ ^]^ bCAII activity is tentatively inhibited in the presence of EAT and recovered after the addition of the target protein owing to the competition between bCAII and the protein for binding to EAT. Re‐activated bCAII then decomposes APmoc‐F(CF_3_)F nanofibers. Overall, integrating the EAT system enables APmoc‐F(CF_3_)F nanofibers/hydrogels to respond to nonenzymatic proteins (Figure 5d).

Covalent polymer gels (agarose) can be hybridized with supramolecular hydrogels and an EAT molecule to construct soft materials that release proteins in response to nonenzymatic proteins (Figure 5b). Confocal images of the orthogonal network of the APmoc‐F(CF_3_)F/agarose composite hydrogel indicated that stimulus‐responsive APmoc‐F(CF_3_)F is retained in the composite hydrogel (Figure 5e). Indeed, HPLC analysis showed that 45% of APmoc‐F(CF_3_)F in the composite hydrogel, which contains bCAII and an EAT molecule (biotin as the ligand for avidin), decomposes in the presence of avidin, a model nonenzymatic protein, while negligible decomposition occurs in the absence of avidin and under a competitive environment for biotin. Notably, the composite hydrogel maintains its gel state even after APmoc‐F(CF_3_)F decomposes, suggesting that the agarose network orthogonally works to retain the structural stability of the composite hydrogel. To realize the controlled release of proteins embedded in the composite hydrogels, we subsequently evaluated the release of entrapped myoglobin (Mb), serving as the model protein. Composite hydrogels containing Mb were immersed in a buffer solution for 3 h, and SDS‐PAGE analysis of the supernatant indicated that 97% of Mb was entrapped in the composite hydrogel. In contrast, only 6.6% of Mb was retained in the single‐component agarose gel, indicating that Mb was entrapped by APmoc‐F(CF_3_)F nanofibers. We then quantified the avidin‐responsive release of embedded Mb from the multicomponent composite hydrogel (Mb/bCAII/EAT⊂APmoc‐F(CF_3_)F/agarose), revealing that 75% of Mb was released in the presence of avidin, whereas only 2.3% of Mb was released in the absence of avidin (Figure 5f,g). We found that this system can be extended to a different combination by designing a corresponding EAT molecule: anti‐DNP antibody as the target protein and RNase A (a promising protein drug for cancer) as the embedded protein (Figure 5h). Our composite system (bCAII/EAT⊂APmoc‐F(CF_3_)F/agarose) can function as an antibody‐responsive RNase A release matrix, which can potentially be used for the release of protein‐based pharmaceuticals controlled by a distinct biomarker protein (antibody). Overall, integrating distinct multiple chemical systems in concert is a promising approach to producing autonomous intelligent soft materials for drug delivery, cell culturing, and regenerative medicine.

### 3.4 Temporal Stimulus Pattern‐Dependent Responses of Synthetic Coacervates

**Figure 6:**
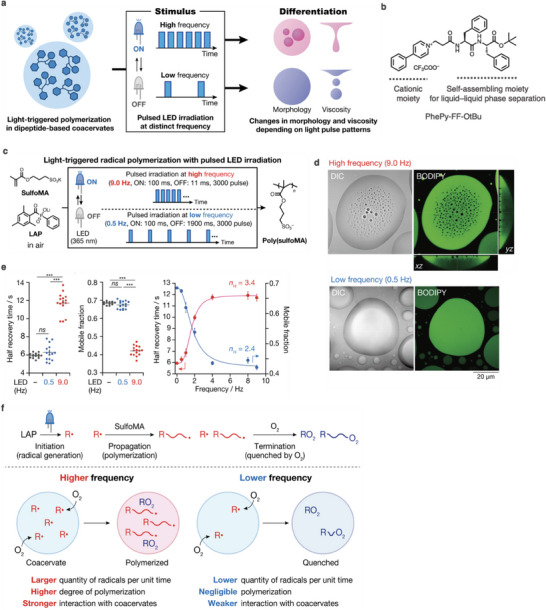
a) Schematic illustration of differentiation of a synthetic dipeptide‐based coacervate in response to temporal stimulus patterns. b) Chemical structure of a cationic dipeptide derivative, PhePy‐FF‐OtBu, for liquid–liquid phase separation. c) Scheme for light‐triggered radical polymerization under pulsed LED irradiation. d) Confocal images of the coacervates after light irradiation at (top) 9 Hz and (bottom) 0.5 Hz. e) (left) Half‐recovery time and (middle) mobile fraction (black) without and with (red) 9 Hz and (blue) 0.5 Hz irradiation. (Right) Frequency‐dependence curves of (red) half‐recovery time and (blue) mobile fraction. f) Plausible mechanism of temporal pattern‐dependent response.

Living systems can decode static signals as well as temporally dynamic signals that vary in frequency and duration into different phenotypic responses.^[^ ^5^, ^97^ ^]^ For example, neural progenitor cells maintain their multipotent state under the oscillatory expression of three different types of transcription factors, while the cells differentiate into three distinct cell types (neurons, astrocytes, and microglia) when oscillatory expression changes to a sustained expression pattern.^[^ ^98^ ^]^ To date, a wide variety of synthetic supramolecules that are responsive to static stimuli have been reported, whereas synthetic systems that can decode the temporal patterns of dynamic signals are rare. In 2022, we reported the differentiation of a synthetic dipeptide‐based coacervate in response to the temporal pattern of a stimulus (**Figure** **6**a).^[^ ^53^ ^]^ The key to achieving this outcome is the construction of a coacervate using a synthetic dipeptide comprising a self‐assembling FF‐OtBu core and a cationic phenylpyridinium group at the *N*‐terminus (PhePy‐FF‐OtBu) to accommodate both light‐triggered radical polymerization and its competitive reaction (Figure 6b). PhePy‐FF‐OtBu coacervate droplets can absorb anionic molecules through electrostatic interactions. The coacervate serves as a unique reaction environment for the light‐triggered radical polymerization of an anionic methacrylate monomer (sulfoMA) in the presence of an anionic initiator (LAP) (Figure 6c). The coacervate environment and temporal light pattern affect the efficiency of radical polymerization, namely, monomer consumption and the degree of polymerization (DP*_n_*), which is conducted in a competitive environment with molecular oxygen. Additionally, the produced anionic polymers interact with the cationic coacervate droplets through multivalent electrostatic interactions to modify the coacervate property. Radical polymerization was triggered by irradiating the coacervate of PhePy‐FF‐OtBu, including sulfoMA and LAP, with dual‐frequency pulsed light (9 and 0.5 Hz) in the air. ^1^H nuclear magnetic resonance (NMR) and size‐exclusion chromatography measurements confirmed that radical polymerization occurs under 9 Hz pulsed‐light irradiation (monomer conversion: 22 ± 2%, DP_n_: 63 ± 5), while negligible polymerization proceeds under 0.5 Hz pulsed‐light irradiation. As a control experiment, radical polymerization was triggered with 0.5 Hz pulsed‐light irradiation under Ar (monomer conversion: 47 ± 9%, DP_n_: 120 ± 30), suggesting that competition between sulfoMA monomers and O_2_ for the reactive radical species is crucial for temporal pattern‐dependent polymerization. Remarkably, coacervate droplets transform into multiphase droplets (coacervate‐in‐coacervate droplets) under 9 Hz pulsed‐light irradiation, while negligible changes occur under 0.5 Hz pulsed‐light irradiation (Figure 6d). Fluorescence recovery after photobleaching (FRAP) analysis revealed that both the mobile fraction and half recovery time after 9 Hz irradiation are significantly different from those of the nonirradiated and 0.5 Hz irradiated samples. Moreover, the values suggested that the inner environment of the coacervate droplets becomes gel‐like after 9 Hz irradiation (Figure 6e). Collectively, the results indicated that the morphology and physical properties of dipeptide‐based coacervates can differentiate in response to temporally distinct pulsed‐light patterns. Furthermore, in‐depth FRAP analysis indicated that the ultrasensitive responses of both the mobile fraction and half recovery time to the irradiation frequency can be characterized by a sigmoid function with a Hill coefficient of 3.4 and 2.4, respectively.^[^ ^99^, ^100^, ^101^ ^]^ A plausible mechanism of this ultrasensitive response is as follows: The threshold amount of reactive radical species produced at the lower frequency (0.5 Hz) is quenched by molecular oxygen diffused from the air, whereas the large amount of radicals produced at the higher frequency (9 Hz) is not completely quenched by molecular oxygen, which allows radical polymerization to proceed (Figure 6f). Overall, cell‐like responses to temporal stimulus patterns emerge by combining the polymer formation reaction and the polymer inhibition reaction involving molecular oxygen diffusion into the viscous coacervate layer.

**Figure 7:**
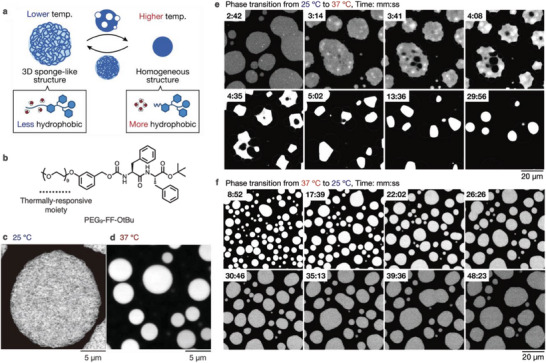
a) Schematic illustration of pathway‐dependent thermally responsive phase transition of a coacervate. b) Chemical structure of the thermally‐responsive dipeptide derivative. c, d) Confocal images of coacervates at (c) the lower temperature phase (25 °C) and (d) the higher temperature phase (37 °C). e, f) Time‐lapse confocal images of thermally‐responsive structural changes of the coacervate (e) from 25 to 37 °C and (f) from 37 and 25 °C.

Stimulus‐responsive coacervates are promising for creating unique multicomponent systems exhibiting dynamic responses.^[^ ^47^, ^102^, ^103^, ^104^, ^105^, ^106^, ^107^, ^108^, ^109^, ^110^ ^]^ We recently found that a coacervate comprising PEG_9_‐FF‐OtBu with a thermally responsive nonaethylene glycol group at the *N*‐terminus of FF‐OtBu undergoes a pathway‐dependent phase transition (**Figure** **7**a,b).^[^ ^54^ ^]^ In the low temperature (LT) phase, PEG_9_‐FF‐OtBu forms coacervate droplets with a 3D sponge‐like inner network, whereas in the high temperature (HT) phase, the coacervate droplets transform into a homogenous structure through a coacervate‐to‐coacervate transition (Figure 7c,d). Time‐lapse confocal imaging revealed that the LT‐to‐HT or HT‐to‐LT phase transition produces distinct intermediate structures. In the path from LT to HT, thin‐layer membrane structures bud at the periphery of the coacervate, and vacuoles (possibly filled with buffer solution) form inside the coacervate (Figure 7e). Subsequently, the vacuoles move to the edge of coacervate droplets, and concurrently the inner structure changes from a sponge‐like network to a homogeneous structure. These changes are completed within 5 min. After incubation, the membranes suddenly burst to afford spherical coacervate droplets. In contrast, along the HT‐to‐LT pathway, the inner coacervate structure gradually transforms into a sponge‐like network over 60 min (Figure 7f). These phase transitions can be reasonably explained by water release and uptake of the PEG_9_ moiety in response to the temperature change. The HT‐to‐LT phase transition requires more time than the LT‐to‐HT phase transition to reach a thermally equilibrated state, probably because of the slow water/buffer uptake into the coacervate droplets for hydration of the PEG_9_ chain.

## 4 Spatiotemporal Control of Static/Dynamic Supramolecular Structures

Living organisms are constructed with 3D hierarchical structures spanning a wide range of scales, from biomolecules to organelles, cells, and organs. Importantly, these structures are tightly linked with sophisticated biological functions.^[^ ^1^ ^]^ These hierarchical structures are prepared by dynamically controlling the formation and degradation of biomolecules and their supramolecular assemblies, which are dependent on the interplay among diverse (enzymatic) reactions, protein–protein interactions, and self‐assembly processes in reaction–diffusion systems.^[^ ^6^, ^7^, ^9^ ^]^

Constructed to mimic naturally occurring systems, spatially patterned synthetic supramolecular systems in 2D and 3D undoubtedly have the potential to serve as functional materials for biomedical applications, such as controlled drug release, tissue engineering, and soft robotics. To date, top‐down approaches, including molding, photo‐patterning, and soft 3D printing, have been widely used to fabricate spatially patterned supramolecular soft materials.^[^ ^36^, ^93^ ^]^ Adams and coworkers used photo‐triggered spatial patterning to construct SDN hydrogels comprising light‐responsive and nonresponsive hydrogelators.^[^ ^68^ ^]^ Owing to self‐sorting, localized irradiation of the hydrogel through a photo mask selectively decomposes light‐responsive nanofibers to generate a patterned network. van Esch and colleagues realized hierarchical compartmentalized multicomponent supramolecular hydrogels by self‐sorting at both the monomer and fiber levels. Hydrazone formation in a system comprising water‐soluble hydrazide and neutral and anionic aldehyde derivatives is accompanied by the in situ generation of multicomponent hydrogelators that self‐sort into nanofibers, which further self‐sort into separated microdomains at the several tens of micrometers scale, resulting in hierarchically compartmentalized supramolecular hydrogels.^[^ ^79^ ^]^ Recently emerged bioinspired bottom‐up approaches involving reaction–diffusion, and phase separation have been combined with conventional top‐down methods to enable the spatiotemporal patterning of multicomponent supramolecular systems.^[^ ^7^, ^31^, ^34^, ^83^, ^111^, ^112^, ^113^, ^114^, ^115^, ^116^, ^117^, ^118^, ^119^, ^120^, ^121^ ^]^ For example, van Esch and coworkers reported the spatially controlled formation of supramolecular hydrogels through a combination of reaction–diffusion, and self‐assembly of hydrogelators in a polymer hydrogel matrix.^[^ ^113^ ^]^ In this approach, nonassembling precursors (aldehyde and hydrazine derivatives) diffuse from opposite sides of the inert agar or Ca^2+^‐alginate gel matrix and react at the crossing front to produce tris‐hydrazone hydrogelators, which subsequently self‐assemble into supramolecular hydrogels. This reaction–diffusion method enables the fabrication of macroscopic hydrogel objects with sizes varying from several hundred micrometers to centimeters. Prins and colleagues demonstrated that ATP‐templated self‐assembled vesicles locally accumulate near the injection point of ATP in an agarose hydrogel matrix.^[^ ^122^ ^]^ In contrast to the conventional reaction–diffusion method (diffusion‐then‐assembly protocol), local self‐assembly of large vesicles with small diffusion coefficients depletes small‐molecule amphiphiles, resulting in the diffusion of amphiphiles toward the injection point along a concentration gradient (assembly‐then‐diffusion protocol). This method allows for progressive local accumulation of vesicles through repetitive ATP injection. Compared with homogeneous gel matrices, the resultant inhomogeneous gel matrix upregulates chemical reactions catalyzed by localized ATP‐templated assemblies. In this section, we highlight the recent results of our studies on the spatial control of multicomponent supramolecular systems through nonequilibrium processes over a wide range of scales, from nanofibers to bulk hydrogels.

### 4.1 Propagating Wave of Supramolecular Nanofibers Undergoing Spatiotemporally Coupled Formation and Degradation

**Figure 8:**
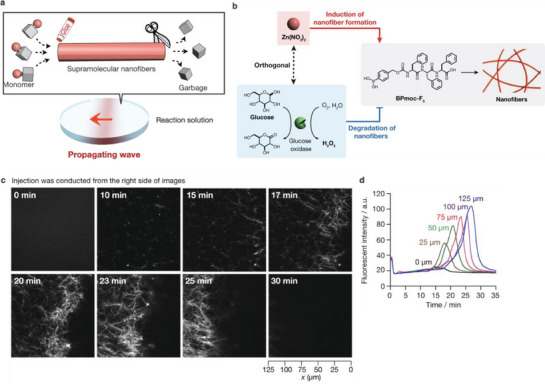
a) Schematic illustration of a propagating wave comprising spatiotemporally‐coupled formation and degradation of supramolecular nanofibers. b) Reaction network for the propagating wave. c) Time‐lapse confocal images of the propagating wave of supramolecular nanofibers. d) Time course of fluorescence intensity at different *x*‐coordinates.

There is a rich variety of dynamic (transient) patterns in living cells. A representative is the propagating wave of actin filaments at lamellipodia, in which polymerization and depolymerization are spatiotemporally controlled by reactions (including ATP hydrolysis), protein–protein interactions, and the diffusion of actin monomers and interacting proteins.^[^ ^2^, ^123^, ^124^ ^]^ We succeeded in generating a propagating wave of synthetic supramolecular nanofibers undergoing spatiotemporally coupled growth and shrinkage (**Figure** **8**a).^[^ ^115^ ^]^

Non‐interfering chemical stimuli were employed to induce nanofibers to undergo stimuli‐induced formation and degradation, thus producing the supramolecular propagating wave (Figure 8b). Zn^2+^ ions were incorporated into BPmoc‐F_3_ through coordination bonding with the carboxylate group of BPmoc‐F_3_ to enhance intermolecular interactions and reduce charge repulsion, thus promoting the formation of BPmoc‐F_3_ nanofibers. As the degradation stimulus, we selected H_2_O_2_ to react with the phenylboronic acid group to convert BPmoc‐F_3_ into a more hydrophilic triphenylalanine peptide, resulting in nanofiber decomposition. H_2_O_2_ is produced in situ through an enzymatic reaction of glucose oxidase (GOx) and glucose. It is known that Zn^2+^ ions do not affect the activity of GOx, and they do not react with H_2_O_2_. Propagating waves emerge along the concentration gradient of the chemical stimuli (Zn^2+^ ions and glucose) created by injecting these stimuli to one side of a mixture of BPmoc‐F_3_, GOx, and a fluorescent probe. Confocal microscopy was used to visualize the small puncta formed after stimulus addition, the nanofibers growing from one side to the other side of the field of view, and the nanofibers gradually disappearing along the same direction (Figure 8c). The time profile of the fluorescent intensity at distinct *x* coordinates demonstrated that the time of the maximum fluorescent intensity was delayed as the *x*‐coordinate increased, indicating the formation of a propagating wave of supramolecular nanofibers along the concentration gradient of the stimuli (Figure 8d). The average traveling distance and velocity of the wave were estimated to be 340 ± 40 and 54 ± 8 µm min^−1^, and we found that the pushing force was generated by the supramolecular propagating wave, which was determined to be 0.005 pN, from the displacement of microbeads. Moreover, the numerical analysis and simulation based on a reaction–diffusion mechanism provided important insights into the propagating wave: i) The wave velocity is proportional to the formation kinetics of the nanofibers and the diffusion coefficient of the degradation stimulus, ii) the concentration gradient of the degradation stimulus is one of the key parameters for maintaining the propagating wave, and iii) the diffusion coefficient of the supramolecular fibers is small, which is another important factor for generating the propagating wave.

### 4.2 Construction of Interpenetrated and Parallel Self‐Sorting Networks

Similar to natural constructs,^[^ ^4^, ^125^, ^126^, ^127^ ^]^ the SDN of synthetic supramolecular systems may exhibit a variety of elaborate patterns.^[^ ^51^, ^65^, ^70^, ^71^, ^76^, ^79^, ^80^, ^81^, ^119^, ^128^, ^129^, ^130^, ^131^, ^132^, ^133^, ^134^ ^]^ We recently demonstrated that controlling fiber formation and degradation (polymerization/depolymerization) can provide supramolecular hydrogels with two distinct SDN network patterns (interpenetrated and parallel).^[^ ^51^ ^]^ In particular, the seed formation kinetics is a critical factor for the discriminative preparation of the two patterns.

**Figure 9:**
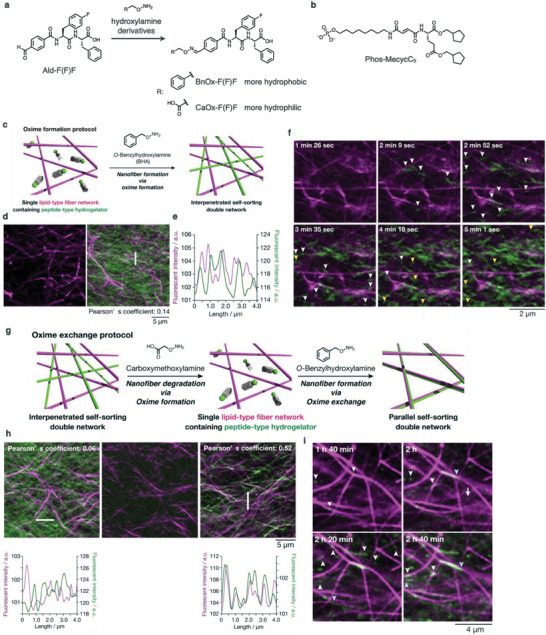
a) Reaction scheme for generation of oxime‐type peptide hydrogelators, BnOx‐F(F)F and CaOx‐F(F)F, from the benzaldehyde‐tethered peptide‐type hydrogelator, Ald‐F(F)F. b) Chemical structure of Phos‐MecycC_5_. c) Scheme of formation of the interpenetrated self‐sorting network through the oxime formation protocol. d) Confocal images before and after the addition of benzylhydroxylamine to a single‐network hydrogel composed of Phos‐MecycC_5_ nanofibers and Ald‐F(F)F. Green: peptide‐type nanofibers, magenta: Phos‐MecycC_5_ nanofibers. e) Line plot analysis along a white line shown in (d). f) Time‐lapse confocal images of the formation of the peptide‐type nanofibers on the surface of the lipid‐type nanofibers (highlighted by white arrows) and in the water layer (highlighted by yellow arrows) g) Scheme of formation of the parallel self‐sorting network through the oxime exchange protocol. h) (top) Confocal images (left) before and after the addition of (middle) carboxylhydroxylamine and (right) benzylhydroxylamine to the SDN hydrogel composed of Phos‐MecycC_5_ and Ald‐F(F)F. Green: peptide‐type nanofibers, magenta: Phos‐MecycC_5_ nanofibers. (bottom) Line plot analyses along white lines. i) Time‐lapse confocal images of the formation of the peptide‐type nanofibers on the surface of the lipid‐type nanofibers (highlighted by white arrows).

We controlled the formation of peptide‐type nanofibers in situ by using dynamic covalent oxime bonding. The reaction of *O*‐benzylhydroxylamine and a peptide‐type hydrogelator with a benzaldehyde group at the *N*‐terminus (Ald‐F(F)F) afforded a hydrophobic benzyloxime‐type hydrogelator (BnOx‐F(F)F) to induce a sol‐to‐gel transition (**Figure** **9**a). Confocal imaging was used to visualize the formation of peptide‐type nanofibers following the addition of *O*‐benzylhydroxylamine to the hydrogel composed of Phos‐MecycC_5_ fibers and Ald‐F(F)F monomers (Figure 9b–d). The line plot analysis confirmed that the formed peptide‐type nanofibers did not overlap with the lipid‐type nanofiber (Figure 9e). The Pearson's correlation coefficient indicated that a negligible correlation existed between these two types of nanofibers, suggesting the formation of an interpenetrated self‐sorting network. Time‐lapse confocal images showed that the peptide‐type nanofibers formed at two distinct nucleation sites: on the surface of lipid‐type nanofibers and at the water layer (Figure 9f). At the initial stage (up to 3 min), short peptide‐type aggregates/fibers selectively nucleated on the surface of the lipid‐type nanofibers. Subsequently, peptide‐type nanofibers appeared on the lipid‐fiber surfaces and within the water layer. These images suggested that nucleation on the lipid‐fiber surface was kinetically favored over nucleation in the water layer.

Confocal microscopic observations also indicated that a decrease in the formation kinetics of BnOx‐F(F)F leads to preferential nucleation on lipid‐type nanofibers, which provides the parallel self‐sorting network. To decelerate the formation kinetics, we employed an oxime‐exchange reaction to transform hydrophilic carboxylate oxime‐type hydrogelator (CaOx‐F(F)F) to BnOx‐F(F)F (Figure 9g). Carboxylic acid hydroxylamine was added to the interpenetrated self‐sorting hydrogel comprising Ald‐F(F)F and Phos‐MecycC_5_ nanofibers, which decomposed the Ald‐F(F)F nanofibers (Figure 9h, left, middle). To this single lipid‐network hydrogel, *O*‐benzylhydroxylamine was subsequently added. As expected, peptide‐type nanofibers were slowly reformed, and these peptide‐type nanofibers were closely aligned but not completely colocalized with the lipid‐type nanofibers (Figure 9h, right). The line plot analysis showed that the peak tops of the peptide‐ and lipid‐type nanofibers were slightly out of alignment, although the peak patterns were similar, suggesting that the parallel self‐sorting network formed. Time‐lapse imaging revealed that 98.6% of the peptide‐type nuclei formed on the surface of the lipid‐type nanofibers (Figure 9i). Moreover, time‐lapse confocal movies revealed that two critical parameters of the formation kinetics of the BnOx‐F(F)F nanofibers differed between the oxime‐formation and oxime‐exchange protocols: the appearance time of short peptide fibers (1.5 min and 1 h 40 min) and the averaged elongation rates (3 ± 3 and 0.06 ± 0.03 µm min^−1^), respectively.

### 4.3 Identification of Four Types of Network Patterns in Supramolecular–Polymer Composite Hydrogels

Differences in the kinetics of self‐assembly may enable the production of multicomponent hydrogels with different network patterns. Recently, we discovered that supramolecular–polymer composite hydrogels can adopt four types of network patterns, which are governed by both the order of network formation and interfiber interactions.^[^ ^119^ ^]^

**Figure 10:**
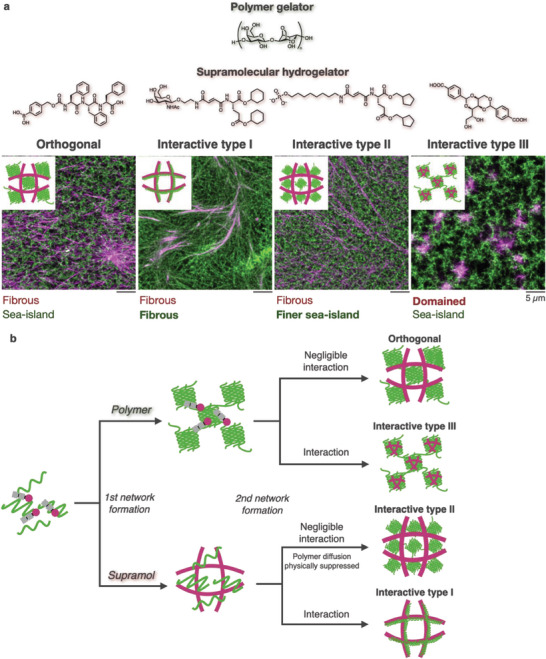
a) (top) Chemical structures of agarose and supramolecular hydrogelators. (bottom) Confocal images of the network structures of the composite hydrogels. Green: agarose, magenta: supramolecular nanofibers. b) Proposed mechanism of the network pattern formation.

We used confocal microscopy to investigate the network structures of composite hydrogels composed of agarose and various supramolecular hydrogelators (**Figure** **10**a). The orthogonal network comprising the supramolecular and agarose networks is morphologically similar to those of the single components, i.e., the fibrous network of the supramolecule and the sea‐island network for agarose. Additionally, three other networks, classified as interactive networks, are characterized by deviations of either the supramolecular or agarose network from the original morphology of the single component. In the case of the GalNAc‐cycC_6_/agarose composite hydrogel, the agarose morphology was altered from the sea‐island network to the fibrous network, while the GalNAc‐cycC_6_ morphology retained the fibrous network. The fibrous agarose network was colocalized with the GalNAc‐cycC_6_ network (termed interactive type I network). In the Phos‐cycC_6_/agarose network, the agarose morphology retained the sea‐island network, albeit with smaller islands and void spaces, while the Phos‐cycC_6_ morphology retained the fibrous network (termed interactive type II network). The DBS‐COOH/agarose composite hydrogel, prepared by decreasing the pH, also adopted the interactive type III network. In this hydrogel, the supramolecular network changed into a heterogeneously distributed domain structure (in the absence of agarose, DBS‐COOH formed the fibrous network, similar to the other supramolecular hydrogelators), while the agarose morphology retained the sea‐island network. The central region of the supramolecular domains overlapped with the island region of the agarose network, indicating that the agarose network served as the nucleation site of DBS‐COOH.

Time‐lapse imaging of the formation processes unveiled two factors governing the generation of the network patterns: the order of network formation and interfiber interactions (Figure 10b). When the agarose network forms first, the self‐assembly of LMW hydrogelators is affected by the agarose network. If there are negligible interactions between the supramolecule and agarose, the supramolecule forms an independent network without interference from agarose, resulting in the orthogonal network. If LMW hydrogelators and agarose interact with each other, the interactive type III network forms because the nucleation and growth of supramolecular nanofibers preferentially occur in the island domain of the agarose network. When supramolecular fiber formation is faster than agarose network formation, the supramolecular network impacts the diffusion and network of agarose. If the interaction between the supramolecule and agarose is minimal, polymer diffusion is physically inhibited by the supramolecular network to afford the interactive type II network. In the case of substantial interaction, the interactive type I network is formed through preferential aggregation of the agarose network on the supramolecular nanofibers serving as a scaffold. Therefore, delicate control of the formation kinetics and interfiber interactions is essential to obtain µm‐sized network patterns of multicomponent synthetic hydrogels.

### 4.4 Fracture‐Induced 3D Patterning of Supramolecular–Polymer Composite Hydrogels

**Figure 11:**
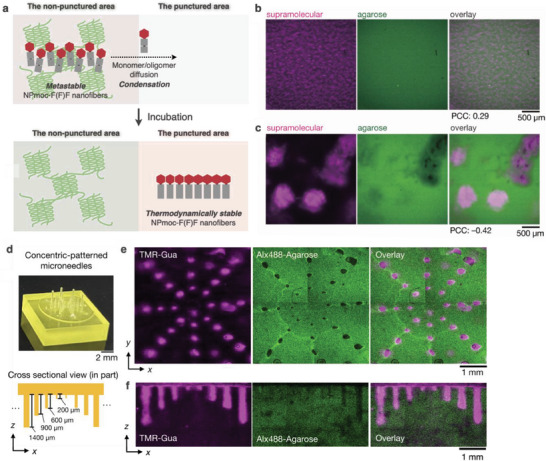
a) Schematic illustration of fracture‐induced patterning of the composite hydrogel. b,c) Confocal images of the composite hydrogel of NPmoc‐F(F)F and agarose b) before and c) after incubation for 24 h. Green: agarose, magenta: NPmoc‐F(F)F nanofibers. d) (top) Photograph of a microneedle stamp with concentric pattern. (bottom) An illustration of the side view of the stamp. e) Confocal images and f) *xz*‐sectional images of the 3D‐patterned composite hydrogel punctured with the concentric‐patterned stamp.

Spatial patterning of multicomponent synthetic hydrogels on the bulk scale (>100 µm) can be constructed by exploiting certain dynamic processes, such as reaction–diffusion and phase separation, during top‐down fabrication.^[^ ^113^, ^119^, ^122^, ^135^, ^136^, ^137^, ^138^, ^139^, ^140^ ^]^ For example, we created fractures to induce network remodeling of supramolecular–polymer composite hydrogels in 3D (**Figure** **11**a).^[^ ^119^ ^]^

By chance, we discovered that a composite hydrogel spontaneously alters its network on the several‐hundred‐micrometer scale during incubation. As‐prepared NPmoc‐F(F)F/agarose hydrogel forms a homogeneously distributed network, and the supramolecular (NPmoc‐F(F)F) network gradually transforms into heterogeneously segregated domains with diameters of approximately 500 µm after room‐temperature incubation (Figure 11b,c). Concurrently, the agarose network also becomes heterogeneously distributed, impinging the supramolecular network domains. These observations suggested that autonomous network remodeling is similar to phase separation in an aqueous two‐phase system. Circular dichroism (CD) spectra indicated that the packing structure of NPmoc‐F(F)F nanofibers is another factor of network remodeling. Subsequently, we punctured the hydrogel exhibiting autonomous remodeling with 3D‐printed microneedle stamps to induce 3D patterning. For example, using a concentric‐patterned microneedle stamp with needle lengths varying from 200 to 1400 µm, we induced macroscopic patterning in the condensed region of NPmoc‐F(F)F nanofibers to controlled depths (Figure 11d,e). Fracture‐induced network remodeling using micrometer‐precision top‐down techniques may facilitate the spatially programmable patterning of functional multicomponent hydrogels in 2D and 3D.

### 4.5 Light‐Triggered Out‐of‐Equilibrium Patterning of Self‐Sorting Supramolecular Hydrogels

**Figure 12:**
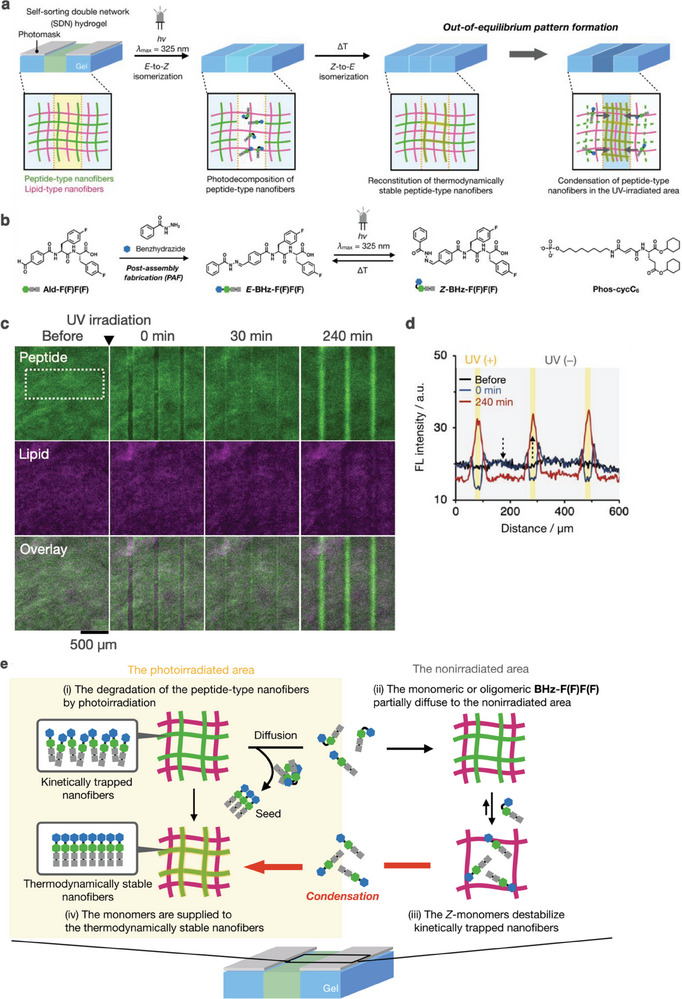
a) Schematic illustration of light‐triggered out‐of‐equilibrium patterning of an SDN hydrogel. b) (left) Scheme for preparation of a light‐responsive peptide‐type hydrogelator and its light‐responsive isomerization. (right) Chemical structure of Phos‐cycC_6_. c) Time‐lapse confocal images before and after light irradiation with a line‐shaped photomask. Green: peptide‐type nanofibers, magenta: lipid‐type nanofibers. d) Line plot analysis along a white box shown in (c). e) Plausible mechanism of light‐triggered out‐of‐equilibrium pattering of the SDN hydrogel.

Photo‐patterning of materials is widely used for top‐down fabrication.^[^ ^36^, ^52^, ^68^, ^69^, ^83^, ^137^, ^141^, ^142^, ^143^, ^144^, ^145^, ^146^, ^147^, ^148^, ^149^, ^150^, ^151^, ^152^, ^153^, ^154^, ^155^, ^156^, ^157^ ^]^ We achieved light‐triggered out‐of‐equilibrium patterning of an SDN hydrogel comprising photo‐responsive peptide‐type and lipid‐type hydrogelators by inducing reaction–diffusion and seed‐driven fiber formation (**Figure** **12**a).^[^ ^83^ ^]^ This out‐of‐equilibrium patterning proceeds through the spatial condensation of peptide‐type nanofibers in the photo‐irradiated areas and concurrent nanofiber depletion in the nonirradiated areas.

To prepare a photo‐responsive peptide‐type hydrogelator, we attached the light‐switchable benzoylhydrazone group to the *N*‐terminus of BHz‐F(F)F(F) (Figure 12b).^[^ ^157^, ^158^, ^159^ ^]^ The *E*‐isomer of the benzoylhydrazone group isomerizes into the *Z*‐isomer upon UV‐light irradiation and thermally reverts to the *E*‐isomer during incubation at room temperature. The *Z*‐isomer disturbs the packing mode of the peptide nanofibers, and thus the nanofibers destabilize upon light irradiation and thermally reform. The photo‐responsive SDN hydrogel was prepared using the PAF protocol. Specifically, benzhydrazide was added to the SDN hydrogel comprising Ald‐F(F)F(F) and Phos‐cycC_6_ to attach the benzoylhydrazone group to the peptide nanofibers. Time‐lapse confocal microscopic images were obtained with low‐magnification objectives before and after localized light irradiation (*λ*_max_: 325 nm) through a line‐shaped photomask (line width, 20 µm; interval, 180 µm) (Figure 12c). Photo‐irradiation generates dark lines in the peptide‐fiber channel but not in the lipid–fiber channel, indicating that the peptide nanofibers are selectively decomposed. Line plot analysis showed that the fluorescence of peptide nanofibers in the irradiated area gradually strengthens during the thermal incubation, and the fluorescence of peptide nanofibers in the nonirradiated area concurrently weakens (Figure 12d). High‐magnification confocal laser scanning microscopy images showed that the numerous BHz‐F(F)F(F) nanofibers in the photo‐irradiated area are regenerated close to peptide aggregates used as seeds. Therefore, the photo‐triggered out‐of‐equilibrium pattern arises from the condensation of peptide nanofibers regenerated in the photo‐irradiated areas where peptide monomers are supplied from the nonirradiated area by diffusion (Figure 12e). CD spectroscopic analysis revealed that the as‐prepared BHz‐F(F)F(F) nanofibers are in the metastable state and the nanofibers reformed after photo‐irradiation are in the thermodynamically stable state.

We applied this out‐of‐equilibrium patterning to create unique photo‐triggered patterns, which are inaccessible by conventional photofabrication of materials. For example, a double‐line pattern was obtained upon consecutive light irradiation through two distinct alignments of a one‐line photomask (line width: 40 µm) and a unique grid‐like pattern. Additionally, spatial patterning of non‐photo‐responsive proteins is possible if the proteins interact with the BHz‐F(F)F(F) nanofibers. These results highlight the power of integrating photochemical response, kinetic trapping, and diffusion‐controlled self‐assembly in multicomponent supramolecular materials, which can facilitate the rational design of synthetic materials with spatiotemporally controlled hierarchical structures and functions.

## 5 Conclusion and Future Perspective

**Figure 13:**
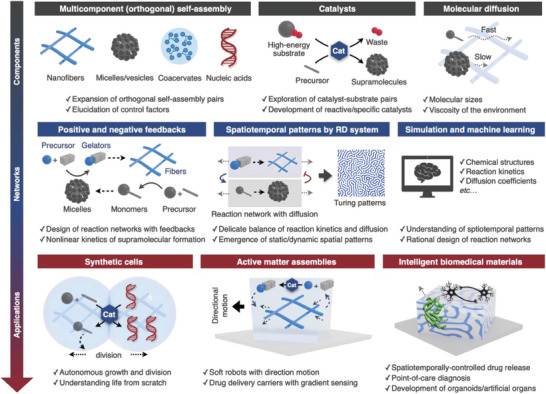
Roadmap toward potential applications of multicomponent non‐/out‐of‐equilibrium dynamic systems. RD: reaction‐diffusion.

In this perspective, we briefly describe our recent achievements in fabricating multicomponent supramolecular systems exhibiting cell‐like responses and hierarchical structures by integrating multiple supramolecules and functional molecules in an orthogonal and/or cooperative manner. With these methods, other researchers also constructed sophisticated supramolecular assemblies exhibiting unique dynamic behaviors, including dynamic instability and oscillation.^[^ ^160^, ^161^, ^162^, ^163^, ^164^, ^165^, ^166^, ^167^, ^168^, ^169^, ^170^, ^171^, ^172^, ^173^, ^174^, ^175^ ^]^ For example, van Esch and coworkers demonstrated the dissipative self‐assembly of a supramolecular hydrogel driven by consuming a chemical fuel.^[^ ^162^, ^176^ ^]^ Methylation of the carboxylate of a hydrogelator precursor by dimethyl sulfate (as the chemical fuel) coupled with ester hydrolysis led to transient self‐assembly of the active methylated hydrogelator. According to time‐lapse confocal images, at the microscopic level, some fibers stochastically collapsed from their tips, while other fibers both grew and shrank simultaneously over a certain period, which is similar to the dynamic instability of microtubules. Hermans and colleagues induced oscillation of a supramolecular polymer composed of a perylene diimide derivative.^[^ ^165^ ^]^ This oscillator was driven by a combination of positive feedback (nucleation–elongation–fragmentation) and negative feedback (size‐dependent depolymerization) under the continuous addition of a reductant as the chemical fuel. Fletcher and coworkers constructed a chemically fueled micelle oscillator using thiol‐disulfide chemistry coupled with the autocatalytic formation and degradation of the active amphiphile in a well‐stirred biphasic setup.^[^ ^173^ ^]^ Otto and coworkers developed a light‐driven protometabolic and eco‐evolutional system of synthetic self‐replicating molecules that can recruit and activate photocatalysts.^[^ ^169^, ^175^ ^]^ These systems rely on the positive feedback loop involving macrocyclic precursors produced by the photocatalytic oxidation of constituents. Exploiting the interplay among multiple functional supramolecules is a promising strategy for the development of next‐generation soft supramolecular materials that are responsive to various stimuli. However, such a systems chemistry approach is in the early stage of development. **Figure** **13** shows a potential roadmap toward cell‐like soft materials, including the development of synthetic cells, active matter assemblies, and intelligent biomedical materials. It is worth noting that in certain areas, the molecular design guidelines and self‐assembly strategies still remain limited: i) expanding multicomponent orthogonal self‐assembly pairs and uncovering control factors; ii) exploring multiple synthetic pairs of high‐energy substrates and catalysts with high reactivity and substrate specificity; iii) designing elaborate reaction networks for nonlinear reaction kinetics; iv) controlling the balance between the reaction kinetics and diffusion rates; and v) understanding and rationally designing non‐/out‐of‐equilibrium phenomena by numerical simulation and machine learning. Therefore, future investigations can extend the variety of supramolecules, functional molecules, and (catalytic) chemical reactions for this approach by focusing on the following directions: i) incorporating DNA/RNA circuits with orthogonality and nonlinear reaction kinetics that can be rationally designed;^[^ ^177^, ^178^, ^179^, ^180^, ^181^, ^182^ ^]^ ii) spatially and temporally segregating interfering chemical reactions by compartmentalizing with vesicles and coacervates;^[^ ^26^, ^27^, ^28^, ^183^, ^184^, ^185^, ^186^ ^]^ and iii) using rapidly developed orthogonal reaction systems for peptide and protein labeling and biomolecule sensing in the field of chemical biology.^[^ ^187^, ^188^, ^189^, ^190^, ^191^, ^192^, ^193^, ^194^, ^195^, ^196^ ^]^ Because their physicochemical properties and functions can be precisely controlled in time and space, autonomously active soft materials with cell‐like responses and spatially controlled structures are promising candidates for a variety of applications, including synthesis of artificial cells having focused functions, soft robotics with directional motion, point‐of‐care disease diagnosis, and tissue regeneration.

## Conflict of Interest

The authors declare no conflict of interest.

## References

[1] B. Alberts , Molecular Biology of the Cell, Garland Science, New York, 2017

[2] L. Blanchoin , R. Boujemaa‐Paterski , C. Sykes , J. Plastino , Physiol. Rev. 2014, 94, 235. 24382887 10.1152/physrev.00018.2013

[3] N. B. Gudimchuk , J. R Mcintosh , Nat. Rev. Mol. Cell Biol. 2021, 22, 777. 34408299 10.1038/s41580-021-00399-x

[4] M. Dogterom , G. H. Koenderink , Nat. Rev. Mol. Cell Biol. 2019, 20, 38. 30323238 10.1038/s41580-018-0067-1

[5] B. N. Kholodenko , J. F. Hancock , W. Kolch , Nat. Rev. Mol. Cell Biol. 2010, 11, 414. 20495582 10.1038/nrm2901 PMC2977972

[6] A. M. Turing , Phil. Trans. R. Soc. B 1952, 237, 37.

[7] S. Soh , M. Byrska , K. Kandere‐Grzybowska , B. A. Grzybowski , Angew. Chem. Int. Ed. 2010, 49, 4170. 10.1002/anie.200905513 PMC3697936 20518023

[8] A. D. Lander , Cell 2007, 128, 245. 17254964 10.1016/j.cell.2007.01.004

[9] S. Kondo , T. Miura , Science 2010, 329, 1616. 20929839 10.1126/science.1179047

[10] S. Kondo , R. Asai , Nature 1995, 376, 765. 24547605 10.1038/376765a0

[11] J. Lutkenhaus , Annu. Rev. Biochem. 2007, 76, 539. 17328675 10.1146/annurev.biochem.75.103004.142652

[12] G. M. Whitesides , B. Grzybowski , Science 2002, 295, 2418. 11923529 10.1126/science.1070821

[13] T. Aida , E. W. Meijer , S. I. Stupp , Science 2012, 335, 813. 22344437 10.1126/science.1205962 PMC3291483

[14] J.‐M. Lehn , Angew. Chem. Int. Ed. 2015, 54, 3276. 10.1002/anie.201409399 25582911

[15] D. J. Cram , Angew. Chem. Int. Ed. 1988, 27, 1009.

[16] J.‐M. Lehn , Angew. Chem. Int. Ed. 1988, 27, 89.

[17] C. J. Pedersen , Angew. Chem. Int. Ed. 1988, 27, 1021.

[18] M. Yoshizawa , J. K. Klosterman , M. Fujita , Angew. Chem. Int. Ed. 2009, 48, 3418. 10.1002/anie.200805340 19391140

[19] C. M. Hong , R. G. Bergman , K. N. Raymond , F. D Toste , Acc. Chem. Res. 2018, 51, 2447. 30272943 10.1021/acs.accounts.8b00328

[20] A. B. Grommet , M. Feller , R. Klajn , Nat. Nanotechnol. 2020, 15, 256. 32303705 10.1038/s41565-020-0652-2

[21] O. M. Yaghi , M. O'keeffe , N. W. Ockwig , H. K. Chae , M. Eddaoudi , J. Kim , Nature 2003, 423, 705. 12802325 10.1038/nature01650

[22] S. Horike , S. Shimomura , S. Kitagawa , Nat. Chem. 2009, 1, 695. 21124356 10.1038/nchem.444

[23] H. G. B. de Jong , H. R. Kruyt , Proc. K. Ned. Akad. Wet. 1929, 32, 849.

[24] A. I. Oparin , Orig. Life 1976, 7, 3. 967402 10.1007/BF01218509

[25] T. Kunitake , Y. Okahata , J. Am. Chem. Soc. 1977, 99, 3860.

[26] A. D. Slootbeek , M. H. I. Van Haren , I. B. A. Smokers , E. Spruijt , Chem. Commun. 2022, 58, 11183. 10.1039/d2cc03541c PMC9536485 36128910

[27] N. Gao , S. Mann , Acc. Chem. Res. 2023, 56, 297. 36625520 10.1021/acs.accounts.2c00696 PMC9910039

[28] A. B. Cook , S. Novosedlik , J. C. M. Van Hest , Acc. Mater. Res. 2023, 4, 287. 37009061 10.1021/accountsmr.2c00239 PMC10043873

[29] T. F. A. De Greef , M. M. J. Smulders , M. Wolffs , A. P. H. J. Schenning , R. P. Sijbesma , E. W. Meijer , Chem. Rev. 2009, 109, 5687. 19769364 10.1021/cr900181u

[30] E. Mattia , S. Otto , Nat. Nanotechnol. 2015, 10, 111. 25652169 10.1038/nnano.2014.337

[31] I. R. Epstein , B. Xu , Nat. Nanotechnol. 2016, 11, 312. 27045215 10.1038/nnano.2016.41

[32] G. Ashkenasy , T. M. Hermans , S. Otto , A. F. Taylor , Chem. Soc. Rev. 2017, 46, 2543. 28418049 10.1039/c7cs00117g

[33] H. Shigemitsu , I. Hamachi , Acc. Chem. Res. 2017, 50, 740. 28252940 10.1021/acs.accounts.7b00070

[34] G. Ragazzon , L. J. Prins , Nat. Nanotechnol. 2018, 13, 882. 30224796 10.1038/s41565-018-0250-8

[35] K. Das , L. Gabrielli , L. J. Prins , Angew. Chem. Int. Ed. 2021, 60, 20120. 10.1002/anie.202100274 PMC8453758 33704885

[36] P. R. A. Chivers , D. K. Smith , Nat. Rev. Mater. 2019, 4, 463.

[37] B. Rieß , R. K. Grötsch , J. Boekhoven , Chem 2020, 6, 552.

[38] I. Aprahamian , ACS Cent. Sci. 2020, 6, 347. 32232135 10.1021/acscentsci.0c00064 PMC7099591

[39] A. Mishra , S. Dhiman , S. J. George , Angew. Chem. Int. Ed. 2021, 60, 2740. 10.1002/anie.202006614 32519456

[40] S. Panja , D. J. Adams , Chem. Soc. Rev. 2021, 50, 5165. 33646219 10.1039/d0cs01166e

[41] S. Borsley , D. A. Leigh , B. M. W. Roberts , Nat. Chem. 2022, 14, 728. 35778564 10.1038/s41557-022-00970-9

[42] P. S. Schwarz , M. Tena‐Solsona , K. Dai , J. Boekhoven , Chem. Commun. 2022, 58, 1284. 10.1039/d1cc06428b 35014639

[43] M. Reches , E. Gazit , Science 2003, 300, 625. 12714741 10.1126/science.1082387

[44] Z. Yang , G. Liang , L. Wang , B. Xu , J. Am. Chem. Soc. 2006, 128, 3038. 16506785 10.1021/ja057412y

[45] Y. Gao , J. Shi , D. Yuan , B. Xu , Nat. Commun. 2012, 3, 1033. 22929790 10.1038/ncomms2040 PMC3521559

[46] C. Yuan , A. Levin , W. Chen , R. Xing , Q. Zou , T. W. Herling , P. K. Challa , T. P. J. Knowles , X. Yan , Angew. Chem., Int. Ed. 2019, 58, 18116. 10.1002/anie.201911782 31617663

[47] M. Abbas , W. P. Lipinski , K. K. Nakashima , W. T. S. Huck , E. Spruijt , Nat. Chem. 2021, 13, 1046. 34645986 10.1038/s41557-021-00788-x

[48] Y. Tang , S. Bera , Y. Yao , J. Zeng , Z. Lao , X. Dong , E. Gazit , G. Wei , Cell Rep. Phys. Sci. 2021, 2, 100579.

[49] M. Ikeda , T. Tanida , T. Yoshii , I. Hamachi , Adv. Mater. 2011, 23, 2819. 21538587 10.1002/adma.201004658

[50] M. Ikeda , T. Tanida , T. Yoshii , K. Kurotani , S. Onogi , K. Urayama , I. Hamachi , Nat. Chem. 2014, 6, 511. 24848237 10.1038/nchem.1937

[51] R. Kubota , K. Nagao , W. Tanaka , R. Matsumura , T. Aoyama , K. Urayama , I. Hamachi , Nat. Commun. 2020, 11, 4100. 32796855 10.1038/s41467-020-17984-x PMC7428048

[52] S. Matsumoto , S. Yamaguchi , S. Ueno , H. Komatsu , M. Ikeda , K. Ishizuka , Y. Iko , K. V. Tabata , H. Aoki , S. Ito , H. Noji , I. Hamachi , Chem. Eur. J. 2008, 14, 3977. 18335444 10.1002/chem.200701904

[53] R. Kubota , S. Torigoe , I. Hamachi , J. Am. Chem. Soc. 2022, 144, 15155. 35943765 10.1021/jacs.2c05101

[54] R. Kubota , T. Hiroi , Y. Ikuta , Y. Liu , I. Hamachi , J. Am. Chem. Soc. 2023, 145, 18316. 37562059 10.1021/jacs.3c03793

[55] A. Heeres , C. Van Der Pol , M. Stuart , A. Friggeri , B. L. Feringa , J. Van Esch , J. Am. Chem. Soc. 2003, 125, 14252. 14624554 10.1021/ja036954h

[56] A. Brizard , M. Stuart , K. Van Bommel , A. Friggeri , M. De Jong , J. Van Esch , Angew. Chem. Int. Ed. 2008, 47, 2063. 10.1002/anie.200704609 18273844

[57] J. Boekhoven , A. M. Brizard , M. C. A. Stuart , L. Florusse , G. Raffy , A. Del Guerzo , J. H. Van Esch , Chem. Sci. 2016, 7, 6021. 30034743 10.1039/c6sc01021k PMC6022170

[58] J. Boekhoven , M. Koot , T. A. Wezendonk , R. Eelkema , J. H. Van Esch , J. Am. Chem. Soc. 2012, 134, 12908. 22823592 10.1021/ja3051876

[59] A. Wada , S.‐I. Tamaru , M. Ikeda , I. Hamachi , J. Am. Chem. Soc. 2009, 131, 5321. 19351208 10.1021/ja900500j

[60] M. Ikeda , T. Yoshii , T. Matsui , T. Tanida , H. Komatsu , I. Hamachi , J. Am. Chem. Soc. 2011, 133, 1670. 21247163 10.1021/ja109692z

[61] M. M. Safont‐Sempere , G. Fernández , F. Würthner , Chem. Rev. 2011, 111, 5784. 21846150 10.1021/cr100357h

[62] L. E. Buerkle , S. J. Rowan , Chem. Soc. Rev. 2012, 41, 6089. 22677951 10.1039/c2cs35106d

[63] J. Raeburn , D. J. Adams , Chem. Commun. 2015, 51, 5170. 10.1039/c4cc08626k 25476555

[64] E. R. Draper , D. J. Adams , Chem. Soc. Rev. 2018, 47, 3395. 29419826 10.1039/c7cs00804j

[65] K. Sugiyasu , S.‐I. Kawano , N. Fujita , S. Shinkai , Chem. Mater. 2008, 20, 2863.

[66] M. R. Molla , A. Das , S. Ghosh , Chem. Eur. J. 2010, 16, 10084. 20583059 10.1002/chem.201000596

[67] K. L. Morris , L. Chen , J. Raeburn , O. R. Sellick , P. Cotanda , A. Paul , P. C. Griffiths , S. M. King , R. K. O'reilly , L. C. Serpell , D. J. Adams , Nat. Commun. 2013, 4, 1480. 23403581 10.1038/ncomms2499

[68] E. R. Draper , E. G. B. Eden , T. O. Mcdonald , D. J. Adams , Nat. Chem. 2015, 7, 848. 26391086 10.1038/nchem.2347

[69] D. J. Cornwell , O. J. Daubney , D. K. Smith , J. Am. Chem. Soc. 2015, 137, 15486. 26646708 10.1021/jacs.5b09691

[70] S. Prasanthkumar , S. Ghosh , V. C. Nair , A. Saeki , S. Seki , A. Ajayaghosh , Angew. Chem. Int. Ed. 2015, 54, 946. 10.1002/anie.201408831 25430809

[71] E. R. Draper , J. R. Lee , M. Wallace , F. Jäckel , A. J. Cowan , D. J. Adams , Chem. Sci. 2016, 7, 6499. 28451108 10.1039/c6sc02644c PMC5355952

[72] N. Singh , K. Zhang , C. A. Angulo‐Pachón , E. Mendes , J. H. Van Esch , B. Escuder , Chem. Sci. 2016, 7, 5568. 30034697 10.1039/c6sc01268j PMC6021788

[73] S. Onogi , H. Shigemitsu , T. Yoshii , T. Tanida , M. Ikeda , R. Kubota , I. Hamachi , Nat. Chem. 2016, 8, 743. 27442279 10.1038/nchem.2526

[74] A. Sarkar , S. Dhiman , A. Chalishazar , S. J. George , Angew. Chem. Int. Ed. 2017, 56, 13767. 10.1002/anie.201708267 28892232

[75] R. Kubota , S. Liu , H. Shigemitsu , K. Nakamura , W. Tanaka , M. Ikeda , I. Hamachi , Bioconjug. Chem. 2018, 29, 2058. 29742348 10.1021/acs.bioconjchem.8b00260

[76] E. R. Cross , S. Sproules , R. Schweins , E. R. Draper , D. J. Adams , J. Am. Chem. Soc. 2018, 140, 8667. 29944359 10.1021/jacs.8b05359

[77] H. Shigemitsu , T. Fujisaku , W. Tanaka , R. Kubota , S. Minami , K. Urayama , I. Hamachi , Nat. Nanotechnol. 2018, 13, 165. 29311611 10.1038/s41565-017-0026-6

[78] W. Tanaka , H. Shigemitsu , T. Fujisaku , R. Kubota , S. Minami , K. Urayama , I. Hamachi , J. Am. Chem. Soc. 2019, 141, 4997. 30835456 10.1021/jacs.9b00715

[79] Y. Wang , M. Lovrak , Q. Liu , C. Maity , V. A. A. Le Sage , X. Guo , R. Eelkema , J. H. Van Esch , J. Am. Chem. Soc. 2019, 141, 2847. 30563317 10.1021/jacs.8b09596 PMC6385057

[80] Y. Wang , R. M. De Kruijff , M. Lovrak , X. Guo , R. Eelkema , J. H. Van Esch , Angew. Chem. Int. Ed. 2019, 58, 3800. 10.1002/anie.201812412 30589169

[81] S. L. Higashi , A. Shibata , Y. Kitamura , K. M. Hirosawa , K. G. N. Suzuki , K. Matsuura , M. Ikeda , Chem. Eur. J 2019, 25, 11955. 31268200 10.1002/chem.201902421

[82] A. Sarkar , R. Sasmal , C. Empereur‐Mot , D. Bochicchio , S. V. K. Kompella , K. Sharma , S. Dhiman , B. Sundaram , S. S. Agasti , G. M. Pavan , S. J. George , J. Am. Chem. Soc. 2020, 142, 7606. 32233467 10.1021/jacs.0c01822

[83] K. Nakamura , W. Tanaka , K. Sada , R. Kubota , T. Aoyama , K. Urayama , I. Hamachi , J. Am. Chem. Soc. 2021, 143, 19532. 34767720 10.1021/jacs.1c09172

[84] N. Singh , A. Lopez‐Acosta , G. J. M. Formon , T. M. Hermans , J. Am. Chem. Soc. 2022, 144, 410. 34932352 10.1021/jacs.1c10282

[85] R. Kubota , K. Nakamura , S. Torigoe , I. Hamachi , ChemistryOpen 2020, 9, 67. 31988842 10.1002/open.201900328 PMC6967000

[86] R. Kubota , W. Tanaka , I. Hamachi , Chem. Rev. 2021, 121, 14281. 33942610 10.1021/acs.chemrev.0c01334

[87] T. Miyata , N. Asami , T. Uragami , Nature 1999, 399, 766. 10391240 10.1038/21619

[88] H. Yang , H. Liu , H. Kang , W. Tan , J. Am. Chem. Soc. 2008, 130, 6320. 18444626 10.1021/ja801339w PMC2757630

[89] W. Bai , N. A. Gariano , D. A. Spivak , J. Am. Chem. Soc. 2013, 135, 6977. 23596978 10.1021/ja400576p

[90] N. Yamaguchi , L. Zhang , B.‐S. Chae , C. S. Palla , E. M. Furst , K. L. Kiick , J. Am. Chem. Soc. 2007, 129, 3040. 17315874 10.1021/ja0680358 PMC2606044

[91] X. Du , J. Zhou , J. Shi , B. Xu , Chem. Rev. 2015, 115, 13165. 26646318 10.1021/acs.chemrev.5b00299 PMC4936198

[92] M. J. Webber , E. A. Appel , E. W. Meijer , R. Langer , Nat. Mater. 2016, 15, 13. 26681596 10.1038/nmat4474

[93] Y. S Zhang , A. Khademhosseini , Science 2017, 356, eaaf3627. 28473537 10.1126/science.aaf3627 PMC5841082

[94] F. Sheehan , D. Sementa , A. Jain , M. Kumar , M. Tayarani‐Najjaran , D. Kroiss , R. V. Ulijn , Chem. Rev. 2021, 121, 13869. 34519481 10.1021/acs.chemrev.1c00089

[95] H. Shigemitsu , R. Kubota , K. Nakamura , T. Matsuzaki , S. Minami , T. Aoyama , K. Urayama , I. Hamachi , Nat. Commun. 2020, 11, 3859. 32737298 10.1038/s41467-020-17698-0 PMC7395795

[96] C.‐W. Wang , W.‐T. Yu , H.‐P. Lai , B.‐Y. Lee , R.‐C. Gao , K.‐T. Tan , Anal. Chem. 2015, 87, 4231. 25811916 10.1021/ac504398g

[97] J. E. Purvis , G. Lahav , Cell 2013, 152, 945. 23452846 10.1016/j.cell.2013.02.005 PMC3707615

[98] I. Imayoshi , A. Isomura , Y. Harima , K. Kawaguchi , H. Kori , H. Miyachi , T. Fujiwara , F. Ishidate , R. Kageyama , Science 2013, 342, 1203. 24179156 10.1126/science.1242366

[99] S. Y. Kim , J. E. Ferrell Jr , Cell 2007, 128, 1133. 17382882 10.1016/j.cell.2007.01.039

[100] J. E. Ferrell Jr , S. H. Ha , Trends Biochem. Sci. 2014, 39, 556. 25440716 10.1016/j.tibs.2014.09.003 PMC4435807

[101] M. Teders , A. A. Pogodaev , G. Bojanov , W. T. S. Huck , J. Am. Chem. Soc. 2021, 143, 5709. 33844531 10.1021/jacs.0c12956 PMC8154525

[102] W. M. Aumiller , C. D. Keating , Nat. Chem. 2016, 8, 129. 26791895 10.1038/nchem.2414

[103] Y. Yin , L. Niu , X. Zhu , M. Zhao , Z. Zhang , S. Mann , D. Liang , Nat. Commun. 2016, 7, 10658. 26876162 10.1038/ncomms10658 PMC4756681

[104] Y. Qiao , M. Li , R. Booth , S. Mann , Nat. Chem. 2017, 9, 110. 28282044 10.1038/nchem.2617

[105] N. Martin , J.‐P. Douliez , Y. Qiao , R. Booth , M. Li , S. Mann , Nat. Commun. 2018, 9, 3652. 30194369 10.1038/s41467-018-06087-3 PMC6128866

[106] A. Bhattacharya , H. Niederholtmeyer , K. A. Podolsky , R. Bhattacharya , J.‐J. Song , R. J. Brea , C.‐H. Tsai , S. K. Sinha , N. K. Devaraj , Proc. Natl. Acad. Sci. USA 2020, 117, 18206. 32694212 10.1073/pnas.2004408117 PMC7414067

[107] C. Donau , F. Späth , M. Sosson , B. A. K. Kriebisch , F. Schnitter , M. Tena‐Solsona , H.‐S. Kang , E. Salibi , M. Sattler , H. Mutschler , J. Boekhoven , Nat. Commun. 2020, 11, 5167. 33056997 10.1038/s41467-020-18815-9 PMC7560875

[108] F. Späth , C. Donau , A. M. Bergmann , M. Kränzlein , C. V. Synatschke , B. Rieger , J. Boekhoven , J. Am. Chem. Soc. 2021, 143, 4782. 33750125 10.1021/jacs.1c01148

[109] M. Matsuo , K. Kurihara , Nat. Commun. 2021, 12, 5487. 34561428 10.1038/s41467-021-25530-6 PMC8463549

[110] N. Gao , C. Xu , Z. Yin , M. Li , S. Mann , J. Am. Chem. Soc. 2022, 144, 3855. 35192333 10.1021/jacs.1c11414 PMC9097475

[111] T. Le Saux , R. Plasson , L. Jullien , Chem. Commun. 2014, 50, 6189. 10.1039/c4cc00392f 24681890

[112] J. Raeburn , B. Alston , J. Kroeger , T. O. Mcdonald , J. R. Howse , P. J. Cameron , D. J. Adams , Mater. Horiz. 2014, 1, 241.

[113] M. Lovrak , W. E. J. Hendriksen , C. Maity , S. Mytnyk , V. Van Steijn , R. Eelkema , J. H. Van Esch , Nat. Commun. 2017, 8, 15317. 28580948 10.1038/ncomms15317 PMC5465320

[114] M. Lovrak , W. E. Hendriksen , M. T. Kreutzer , V. Van Steijn , R. Eelkema , J. H. Van Esch , Soft Matter 2019, 15, 4276. 31038130 10.1039/c8sm02588f

[115] R. Kubota , M. Makuta , R. Suzuki , M. Ichikawa , M. Tanaka , I. Hamachi , Nat. Commun. 2020, 11, 3541. 32669562 10.1038/s41467-020-17394-z PMC7363860

[116] L. Schlichter , C. C. Piras , D. K. Smith , Chem. Sci. 2021, 12, 4162. 34163689 10.1039/d0sc06862d PMC8179439

[117] H. S. Cooke , L. Schlichter , C. C. Piras , D. K. Smith , Chem. Sci. 2021, 12, 12156. 34667581 10.1039/d1sc03155d PMC8457394

[118] K. Das , H. Kar , R. Chen , I. Fortunati , C. Ferrante , P. Scrimin , L. Gabrielli , L. J. Prins , J. Am. Chem. Soc. 2023, 145, 898. 36576874 10.1021/jacs.2c09343 PMC9853849

[119] K. Nakamura , R. Kubota , T. Aoyama , K. Urayama , I. Hamachi , Nat. Commun. 2023, 14, 1696. 36973291 10.1038/s41467-023-37412-0 PMC10042874

[120] Y. Cao , L. Gabrielli , D. Frezzato , L. J. Prins , Angew. Chem. Int. Ed. 2023, 62, e202215421. 10.1002/anie.202215421 36420591

[121] P. A. Korevaar , C. N Kaplan , A. Grinthal , R. M. Rust , J. Aizenberg , Nat. Commun. 2020, 11, 386. 31959819 10.1038/s41467-019-14114-0 PMC6971035

[122] R. Chen , K. Das , M. A. Cardona , L. Gabrielli , L. J. Prins , J. Am. Chem. Soc. 2022, 144, 2010. 35061942 10.1021/jacs.1c13504 PMC8815075

[123] M. Krause , A. Gautreau , Nat. Rev. Mol. Cell Biol. 2014, 15, 577. 25145849 10.1038/nrm3861

[124] M. J. Footer , J. W. J. Kerssemakers , J. A. Theriot , M. Dogterom , Proc. Natl. Acad. Sci. USA 2007, 104, 2181. 17277076 10.1073/pnas.0607052104 PMC1892916

[125] F. Huber , A. Boire , M. P. López , G. H. Koenderink , Curr. Opin. Cell Biol. 2015, 32, 39. 25460780 10.1016/j.ceb.2014.10.005

[126] J. K. Mouw , G. Ou , V. M. Weaver , Nat. Rev. Mol. Cell Biol. 2014, 15, 771. 25370693 10.1038/nrm3902 PMC4682873

[127] C. Bonnans , J. Chou , Z. Werb , Nat. Rev. Mol. Cell Biol. 2014, 15, 786. 25415508 10.1038/nrm3904 PMC4316204

[128] W. Zhang , W. Jin , T. Fukushima , A. Saeki , S. Seki , T. Aida , Science 2011, 334, 340. 22021852 10.1126/science.1210369

[129] M. Lista , J. Areephong , N. Sakai , S. Matile , J. Am. Chem. Soc. 2011, 133, 15228. 21678978 10.1021/ja204020p

[130] J. López‐Andarias , M. J. Rodriguez , C. Atienza , J. L. López , T. Mikie , S. Casado , S. Seki , J. L. Carrascosa , N. Martín , J. Am. Chem. Soc. 2015, 137, 893. 25530351 10.1021/ja510946c

[131] T. Fukui , M. Takeuchi , K. Sugiyasu , Sci. Rep. 2017, 7, 89. 28546565 10.1038/s41598-017-02524-3 PMC5445073

[132] W. Ji , S. Zhang , S. Yukawa , S. Onomura , T. Sasaki , K. '. Miyazawa , Y. Zhang , Angew. Chem., Int. Ed. 2018, 57, 3636. 10.1002/anie.201712575 29411922

[133] L. Su , J. Mosquera , M. F. J. Mabesoone , S. M. C. Schoenmakers , C. Muller , M. E. J. Vleugels , S. Dhiman , S. Wijker , A. R. A. Palmans , E. W. Meijer , Science 2022, 377, 213. 35857543 10.1126/science.abn3438

[134] N. Sasaki , J. Kikkawa , Y. Ishii , T. Uchihashi , H. Imamura , M. Takeuchi , K. Sugiyasu , Nat. Chem. 2023, 15, 922. 37264101 10.1038/s41557-023-01216-y

[135] H. Komatsu , S. Tsukiji , M. Ikeda , I. Hamachi , Chem. Asian J. 2011, 6, 2368. 21721133 10.1002/asia.201100134

[136] D. Kiriya , M. Ikeda , H. Onoe , M. Takinoue , H. Komatsu , Y. Shimoyama , I. Hamachi , S. Takeuchi , Angew. Chem. Int. Ed. 2012, 51, 1553. 10.1002/anie.201104043 22086540

[137] P. R. A. Chivers , D. K. Smith , Chem. Sci. 2017, 8, 7218. 29081954 10.1039/c7sc02210g PMC5633784

[138] M. Tena‐Solsona , B. Rieß , R. K. Grötsch , F. C. Löhrer , C. Wanzke , B. Käsdorf , A. R. Bausch , P. Müller‐Buschbaum , O. Lieleg , J. Boekhoven , Nat. Commun. 2017, 8, 15895. 28719591 10.1038/ncomms15895 PMC5520059

[139] T. Matsuda , R. Kawakami , R. Namba , T. Nakajima , J. P. Gong , Science 2019, 363, 504. 30705187 10.1126/science.aau9533

[140] C. C. Piras , P. Slavik , D. K. Smith , Angew. Chem. Int. Ed. 2020, 59, 853. 10.1002/anie.201911404 PMC6973155 31697017

[141] C. M. Soukoulis , M. Wegener , Nat. Photonics 2011, 5, 523.

[142] J. Eastoe , M. Sánchez‐Dominguez , P. Wyatt , R. K. Heenan , Chem. Commun. 2004, 2608. 10.1039/b410158h 15543303

[143] J. J. D. De Jong , P. R Hania , A. Pugzlys , L. N. Lucas , M. De Loos , R. M. Kellogg , B. L. Feringa , K. Duppen , J. H. Van Esch , Angew. Chem. Int. Ed. 2005, 44, 2373. 10.1002/anie.200462500 15761897

[144] S. Matsumoto , S. Yamaguchi , A. Wada , T. Matsui , M. Ikeda , I. Hamachi , Chem. Commun. 2008, 1545. 10.1039/b719004b 18354794

[145] A. M. Kloxin , A. M. Kasko , C. N. Salinas , K. S. Anseth , Science 2009, 324, 59. 19342581 10.1126/science.1169494 PMC2756032

[146] C. A. Deforest , K. S. Anseth , Nat. Chem. 2011, 3, 925. 22109271 10.1038/nchem.1174 PMC3229165

[147] J. Raeburn , T. O. Mcdonald , D. J. Adams , Chem. Commun. 2012, 48, 9355. 10.1039/c2cc34677j 22890605

[148] S. Lee , S. Oh , J. Lee , Y. Malpani , Y.‐S. Jung , B. Kang , J. Y. Lee , K. Ozasa , T. Isoshima , S. Y. Lee , M. Hara , D. Hashizume , J.‐M. Kim , Langmuir 2013, 29, 5869. 23597134 10.1021/la400159m

[149] T. Yoshii , M. Ikeda , I. Hamachi , Angew. Chem., Int. Ed. 2014, 53, 7264. 10.1002/anie.201404158 24866821

[150] J. T. Van Herpt , M. C. A. Stuart , W. R. Browne , B. L. Feringa , Chem. Eur. J. 2014, 20, 3077. 24677510 10.1002/chem.201304064

[151] C. Maity , W. E. Hendriksen , J. H. Van Esch , R. Eelkema , Angew. Chem. Int. Ed. 2015, 54, 998. 10.1002/anie.201409198 25385283

[152] E. R. Draper , R. Schweins , R. Akhtar , P. Groves , V. Chechik , M. A. Zwijnenburg , D. J. Adams , Chem. Mater. 2016, 28, 6336.

[153] D. Oran , S. G. Rodriques , R. Gao , S. Asano , M. A. Skylar‐Scott , F. Chen , P. W. Tillberg , A. H. Marblestone , E. S. Boyden , Science 2018, 362, 1281. 30545883 10.1126/science.aau5119 PMC6423357

[154] S.‐T. Tung , H.‐T. Cheng , A. Inthasot , F.‐C. Hsueh , T.‐J. Gu , P.‐C. Yan , C.‐C. Lai , S.‐H. Chiu , Chem. Eur. J. 2018, 24, 1522. 29226433 10.1002/chem.201705753

[155] M. E. Roth‐Konforti , M. Comune , M. Halperin‐Sternfeld , I. Grigoriants , D. Shabat , L. Adler‐Abramovich , Macromol. Rapid Commun. 2018, 39, e1800588. 30276909 10.1002/marc.201800588

[156] X. Li , J. Fei , Y. Xu , D. Li , T. Yuan , G. Li , C. Wang , J. Li , Angew. Chem. Int. Ed. 2018, 57, 1903. 10.1002/anie.201711547 29280315

[157] E. Weyandt , G. M. Ter Huurne , G. Vantomme , A. J. Markvoort , A. R. A. Palmans , E. W. Meijer , J. Am. Chem. Soc. 2020, 142, 6295. 32167302 10.1021/jacs.0c00858 PMC7118707

[158] D. J. Van Dijken , P. Kovaříček , S. P. Ihrig , S. Hecht , J. Am. Chem. Soc. 2015, 137, 14982. 26580808 10.1021/jacs.5b09519

[159] A. Ryabchun , Q. Li , F. Lancia , I. Aprahamian , N. Katsonis , J. Am. Chem. Soc. 2019, 141, 1196. 30624915 10.1021/jacs.8b11558 PMC6346373

[160] R. Klajn , P. J. Wesson , K. J. M. Bishop , B. A. Grzybowski , Angew. Chem. Int. Ed. 2009, 48, 7035. 10.1002/anie.200901119 19533698

[161] C. G. Pappas , I. R. Sasselli , R. V. Ulijn , Angew. Chem. Int. Ed. 2015, 54, 8119. 10.1002/anie.201500867 26014441

[162] J. Boekhoven , W. E. Hendriksen , G. J. M. Koper , R. Eelkema , J. H. Van Esch , Science 2015, 349, 1075. 26339025 10.1126/science.aac6103

[163] S. Maiti , I. Fortunati , C. Ferrante , P. Scrimin , L. J. Prins , Nat. Chem. 2016, 8, 725. 27325101 10.1038/nchem.2511

[164] T. Ikegami , Y. Kageyama , K. Obara , S. Takeda , Angew. Chem. Int. Ed. 2016, 55, 8239. 10.1002/anie.201600218 27194603

[165] J. Leira‐Iglesias , A. Tassoni , T. Adachi , M. Stich , T. M. Hermans , Nat. Nanotechnol. 2018, 13, 1021. 30323361 10.1038/s41565-018-0270-4

[166] A. Jain , S. Dhiman , A. Dhayani , P. K. Vemula , S. J. George , Nat. Commun. 2019, 10, 450. 30683874 10.1038/s41467-019-08308-9 PMC6347607

[167] S. Bal , K. Das , S. Ahmed , D. Das , Angew. Chem. Int. Ed. 2019, 58, 244. 10.1002/anie.201811749 30395376

[168] S. Panja , B. Dietrich , D. J. Adams , ChemSystemsChem 2020, 2, 190003.

[169] G. Monreal Santiago , K. Liu , W. R. Browne , S. Otto , Nat. Chem. 2020, 12, 603. 32591744 10.1038/s41557-020-0494-4

[170] M. Samperi , B. Bdiri , C. D. Sleet , R. Markus , A. R. Mallia , L. Pérez‐García , D. B. Amabilino , Nat. Chem. 2021, 13, 1200. 34635814 10.1038/s41557-021-00791-2

[171] F. Schnitter , B. Rieß , C. Jandl , J. Boekhoven , Nat. Commun. 2022, 13, 2816. 35595758 10.1038/s41467-022-30424-2 PMC9122941

[172] S. Selmani , E. Schwartz , J. T. Mulvey , H. Wei , A. Grosvirt‐Dramen , W. Gibson , A. I. Hochbaum , J. P. Patterson , R. Ragan , Z. Guan , J. Am. Chem. Soc. 2022, 144, 7844. 35446034 10.1021/jacs.2c01884

[173] M. G. Howlett , A. H. J. Engwerda , R. J. H. Scanes , S. P. Fletcher , Nat. Chem. 2022, 14, 805. 35618766 10.1038/s41557-022-00949-6

[174] D. Barpuzary , P. J. Hurst , J. P. Patterson , Z. Guan , J. Am. Chem. Soc. 2023, 145, 3727. 36746118 10.1021/jacs.2c13140

[175] K. Liu , A. Blokhuis , C. Van Ewijk , A. Kiani , J. Wu , W. H. Roos , S. Otto , Nat. Chem. 2023, 10.1038/s41557-023-01301-2. 37653230

[176] J. Boekhoven , A. M. Brizard , K. N. K. Kowlgi , G. J. M. Koper , R. Eelkema , J. H. Van Esch , Angew. Chem., Int. Ed. 2010, 49, 4825. 10.1002/anie.201001511 20512834

[177] G. Seelig , D. Soloveichik , D. Y. Zhang , E. Winfree , Science 2006, 314, 1585. 17158324 10.1126/science.1132493

[178] L. Qian , E. Winfree , Science 2011, 332, 1196. 21636773 10.1126/science.1200520

[179] A. Padirac , T. Fujii , A. Estévez‐Torres , Y. Rondelez , J. Am. Chem. Soc. 2013, 135, 14586. 23731347 10.1021/ja403584p

[180] G. Gines , A. S. Zadorin , J.‐C. Galas , T. Fujii , A. Estevez‐Torres , Y. Rondelez , Nat. Nanotechnol. 2017, 12, 351. 28135261 10.1038/nnano.2016.299

[181] A. Joesaar , S. Yang , B. Bögels , A. Van Der Linden , P. Pieters , B. V. V. S. P Kumar , N. Dalchau , A. Phillips , S. Mann , T. F. A. De Greef , Nat. Nanotechnol. 2019, 14, 369. 30833694 10.1038/s41565-019-0399-9 PMC6451639

[182] S. Okumura , G. Gines , N. Lobato‐Dauzier , A. Baccouche , R. Deteix , T. Fujii , Y. Rondelez , A. J. Genot , Nature 2022, 610, 496. 36261553 10.1038/s41586-022-05218-7

[183] J. W. Szostak , D. P. Bartel , P. L Luisi , Nature 2001, 409, 387. 11201752 10.1038/35053176

[184] S. S. Mansy , J. P. Schrum , M. Krishnamurthy , S. Tobé , D. A. Treco , J. W. Szostak , Nature 2008, 454, 122. 18528332 10.1038/nature07018 PMC2743009

[185] U. J. Meierhenrich , J.‐J. Filippi , C. Meinert , P. Vierling , J. P. Dworkin , Angew. Chem., Int. Ed. 2010, 49, 3738. 10.1002/anie.200905465 20437432

[186] P. L. Luisi , The Emergence of Life: From Chemical Origins to Synthetic Biology, Cambridge University Press, Cambridge, MA 2016.

[187] B. A Griffin , S. R. Adams , R. Y. Tsien , Science 1998, 281, 269. 9657724 10.1126/science.281.5374.269

[188] B. F. Cravatt , A. T. Wright , J. W. Kozarich , Annu. Rev. Biochem. 2008, 77, 383. 18366325 10.1146/annurev.biochem.75.101304.124125

[189] E. M. Sletten , C. R. Bertozzi , Angew. Chem., Int. Ed. 2009, 48, 6974. 10.1002/anie.200900942 PMC2864149 19714693

[190] C. D. Spicer , B. G. Davis , Nat. Commun. 2014, 5, 4740. 25190082 10.1038/ncomms5740

[191] K. Lang , J. W. Chin , Chem. Rev. 2014, 114, 4764. 24655057 10.1021/cr400355w

[192] L. Xue , I. A. Karpenko , J. Hiblot , K. Johnsson , Nat. Chem. Biol. 2015, 11, 917. 26575238 10.1038/nchembio.1959

[193] K. K. Palaniappan , C. R. Bertozzi , Chem. Rev. 2016, 116, 14277. 27960262 10.1021/acs.chemrev.6b00023 PMC5327817

[194] B. L. Oliveira , Z. Guo , G. J. L. Bernardes , Chem. Soc. Rev. 2017, 46, 4895. 28660957 10.1039/c7cs00184c

[195] S. Han , J. Li , A. Y. Ting , Curr. Opin. Neurobiol. 2018, 50, 17. 29125959 10.1016/j.conb.2017.10.015 PMC6726430

[196] T. Tamura , I. Hamachi , J. Am. Chem. Soc. 2019, 141, 2782. 30592612 10.1021/jacs.8b11747

